# High-Resolution Ion-Flux Imaging of Proton Transport through Graphene|Nafion Membranes

**DOI:** 10.1021/acsnano.1c05872

**Published:** 2022-03-14

**Authors:** Cameron L. Bentley, Minkyung Kang, Saheed Bukola, Stephen E. Creager, Patrick R. Unwin

**Affiliations:** † School of Chemistry, Monash University, Clayton, Victoria 3800, Australia; ‡ Department of Chemistry, University of Warwick, Coventry CV4 7AL, United Kingdom; § Department of Chemistry, Clemson University, Clemson, South Carolina 29634, United States

**Keywords:** scanning electrochemical cell microscopy, SECCM, 2D materials, defects, nanopores

## Abstract

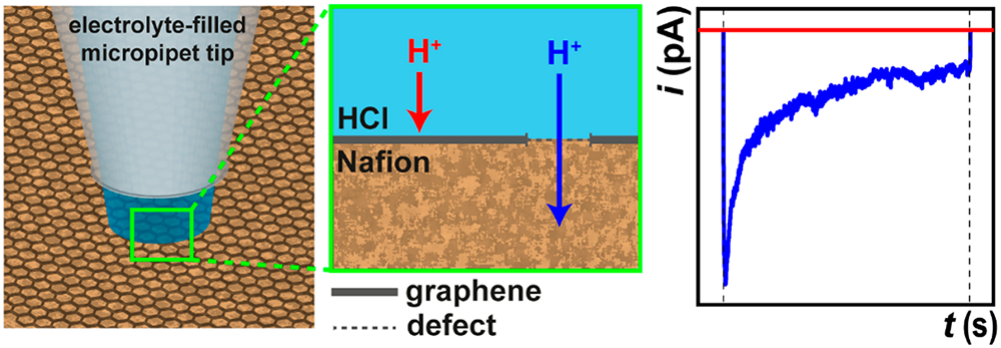

In 2014, it was reported that protons can traverse between aqueous phases separated by nominally pristine monolayer graphene and hexagonal boron nitride (h-BN) films (membranes) under ambient conditions. This intrinsic proton conductivity of the one-atom-thick crystals, with proposed through-plane conduction, challenged the notion that graphene is impermeable to atoms, ions, and molecules. More recent evidence points to a defect-facilitated transport mechanism, analogous to transport through conventional ion-selective membranes based on graphene and h-BN. Herein, local ion-flux imaging is performed on chemical vapor deposition (CVD) graphene|Nafion membranes using an “electrochemical ion (proton) pump cell” mode of scanning electrochemical cell microscopy (SECCM). Targeting regions that are free from visible macroscopic defects (*e.g.*, cracks, holes, *etc.*) and assessing hundreds to thousands of different sites across the graphene surfaces in a typical experiment, we find that most of the CVD graphene|Nafion membrane is impermeable to proton transport, with transmission typically occurring at ≈20–60 localized sites across a ≈0.003 mm^2^ area of the membrane (>5000 measurements total). When localized proton transport occurs, it can be a highly dynamic process, with additional transmission sites “opening” and a small number of sites “closing” under an applied electric field on the seconds time scale. Applying a simple equivalent circuit model of ion transport through a cylindrical nanopore, the local transmission sites are estimated to possess dimensions (radii) on the (sub)nanometer scale, implying that rare atomic defects are responsible for proton conductance. Overall, this work reinforces SECCM as a premier tool for the structure–property mapping of microscopically complex (electro)materials, with the local ion-flux mapping configuration introduced herein being widely applicable for functional membrane characterization and beyond, for example in diagnosing the failure mechanisms of protective surface coatings.

Over the past decade, graphene and related two-dimensional (2D) materials have been increasingly explored as ion-selective membranes for diverse applications ranging from clean energy generation/storage technologies^1^ to water remediation/desalination.^2^ The atomic thickness of these materials, coupled with high mechanical strength, chemical inertness, and tunable surface chemistry has evoked the possibility of “designer” membranes with tailorable properties (**i.e.**, permeance, selectivity, *etc.*).^3,4^ With the exception of protons,^5^ it is generally accepted that selective ion (as well as gas^6^ and DNA^7^) transport through graphene is facilitated by (sub)nanometer-sized pores naturally present at intrinsic defects^8,9^ and/or deliberately introduced by physical (*e.g.*, ion bombardment) or chemical (*e.g.*, ozone treatment and/or oxidative etching) treatment.^9,10^

In 2014, anomalously high proton transport through nominally pristine monolayer graphene and hexagonal boron nitride (h-BN) membranes (prepared by mechanical exfoliation) was reported,^5^ with areal conductivity (*G*/*A*, where *G* is electrical conductance and *A* is area) values of ≈3 and ≈100 mS cm^–2^, respectively, at room temperature (cf. ≈10 S cm^–2^ for hydrated Nafion 212 membrane, 50 μm thick^11^). These *G*/*A* values represented the intrinsic proton conductivity of the studied 2D crystals (*i.e.*, through-plane proton conduction),^5,12^ challenging the widely accepted notion that pristine graphene is impermeable to all atoms, ions, and molecules under ambient conditions.^13,14^ Subsequent studies by several research groups^15−18^ have suggested that selective proton transport may be facilitated at defect sites (naturally occurring^15,16^ or introduced^17,18^) that are likely separate from the sites that facilitate the transport of other ions (*i.e.*, pores in nanoporous graphene, *vide supra*).^8,11,19^

There has been interest in scaling up proton-selective membranes based on graphene and related 2D materials.^11,12,20,21^ For example, a scalable “electrochemical proton pump cell” configuration in which macroscopic (square centimeter scale) graphene sheets produced by chemical vapor deposition (CVD) were deposited onto a commercially available perfluorosulfonic acid polymer (Nafion) film, was reported.^12^ CVD graphene-on-Nafion membranes (referred to as graphene|Nafion, herein) are able to achieve much higher proton transport rates (*e.g.*, *G*/*A* > 10 S cm^–2^),^11^ while maintaining relatively high selectivity (>100× higher *G*/*A* values compared to those of Li^+^, Na^+^, K^+^, Rb^+^, Cs^+^, or NH_4_^+^).^19^ Although it is well-known that CVD graphene possesses a distribution of intrinsic defects (*e.g.*, from atomic vacancies^16^ to nanometer-sized pores,^8^*vide supra*), as yet there is no direct evidence for heterogeneous transmission at particular locations on graphene|Nafion membranes. Conventional Raman spectroscopy lacks the spatial resolution and sensitivity to detect defects in high-quality graphene (*i.e.*, graphene with defect densities below ≈20 μm^–2^ are expected to appear “pristine” in Raman^22^), and although high-resolution microscopy (*e.g.*, transmission electron microscopy, TEM^23^) has sufficient resolution to locally image defects, it is only able to provide a limited view in a macroscopic sense.^8,15^

The extraction of large-scale statistics on local proton transmission through graphene|Nafion requires a high-throughput technique that can directly probe/map ion flux with high spatial resolution over larger areas of the membrane.^24^ Scanning electrochemical cell microscopy (SECCM)^25−27^ stands out as the ideal technique for this application, as it uses a fluidic micropipet/nanopipet probe to carry out local electrochemistry (and ion-conductance measurements, *vide infra*) within a confined region of an electrode surface, with a spatial resolution (down to tens of nanometer^28,29^) defined by the area of meniscus contact. In recent years, SECCM has predominantly been used in conjunction with colocated microscopy/spectroscopy to reveal structure–activity in a diverse range of (electro)materials,^24,30^ including 2D materials such as graphene^31^ and transition metal dichalcogenides, such as MoS_2_,^32−35^ WS_2_,^34^ WSe_2_,^32,36^ MoS_2_/WS_2_ heterostructures,^37^*etc.* However, SECCM with a dual-channel probe^38^ can also make real-time, local ion conductance measurements on any type of surface, regardless of electrical conductivity.^39^ As we show herein, this configuration is crucial to land the meniscus cell on any part of the surface, irrespective of the local proton transmissibility.

In this work, the synchronous electrochemical activity and ion conductance mapping capabilities of SECCM are exploited to probe local proton transmission through a previously reported^11^ graphene|Nafion membrane prepared by a hot-press method. The micropipet probe is deployed as an electrochemical ion (proton) pump cell^40^ to target regions of the graphene|Nafion membrane that are free from macroscopic defects (*e.g.*, cracks), revealing that, in these devices, most of the graphene surface is impermeable to protons, with transmission typically occurring only at ∼20–60 localized sites across a ≈0.003 mm^2^ area. This localized proton transport process can also be highly dynamic, with a few additional transmission sites “opening up” on the seconds time scale when exposed to a proton-driving voltage (from the applied electric field) across the graphene|Nafion membrane. By analogizing localized proton transmission to ion transport through a cylindrical nanopore,^4,41^ we can predict a simple equivalent circuit model in which each site/pore possesses radii on the (sub)nanometer scale and thus may be attributable to the presence of one or more atomic defects in the graphene overlayer. All in all, this work further reinforces the status of SECCM as a premier tool for local ion-flux mapping of microscopically complex (electro)materials.

## Results and Discussion

### Spatially Resolved Proton Conductance Measurements

To investigate local proton transport through graphene, spatially resolved electrochemical measurements were performed on graphene|Nafion membranes using SECCM in the dual-channel configuration. The Nafion 211 membrane (≈25 μm thickness) behaves as both a solid support and a highly conductive proton source/sink (bulk conductivity estimated to be on the order of ∼20–60 mS cm^–1^ under the conditions explored).^42^ The monolayer graphene film (situated on top of the Nafion 211 support) is investigated as a proton-selective membrane. Herein, the graphene|Nafion membrane assembly was fabricated by a hot-press method similar to that used previously,^11,19^ which, as established for the membrane electrode assemblies used in fuel cells,^43^ should ensure intimate interfacial contact between graphene and the protogenic groups in Nafion, allowing for efficient proton transmission through the hydrated sandwich structure. Note that after fabrication of the graphene|Nafion membranes, the quality of the graphene overlayer was assessed *via* SECCM measurements of the FcDM^0/+^ process (FcDM = ferrocenedimethanol). After the tip was positioned, as for the proton conduction measurements (*vide infra*), this redox process was found to be kinetically facile (*i.e.*, electrochemically reversible) in randomly selected spots, confirming the graphene preparation yielded a surface of sufficient quality for electron tunneling (electrochemical) measurements [see Supporting Information (SI), Figure S1].

Nafion is characterized by a complex, humidity-dependent nanostructure, with distinct domains of high and low ionic conductivity, corresponding to the hydrophilic sulfonate groups and hydrophobic fluorocarbon backbone, respectively.^44−46^ Note that these distinct domains are typically on the order of nanometers to tens of nanometers in scale,^44^ which means that the Nafion can effectively be treated as an isotropic proton source/sink (*e.g.*, a liquid electrolyte) on the scale of the SECCM probes (≈micrometer scale) used herein, assuming that intrinsic proton transfer occurs uniformly across the graphene surface *via* a through-plane conduction mechanism (see SI Figure S2a). Indeed, Nafion has previously been used as a graphene support for proton transmission measurements with similarly sized^5^ and larger macroscopic^11,12,19^ devices.

**Figure 1:**
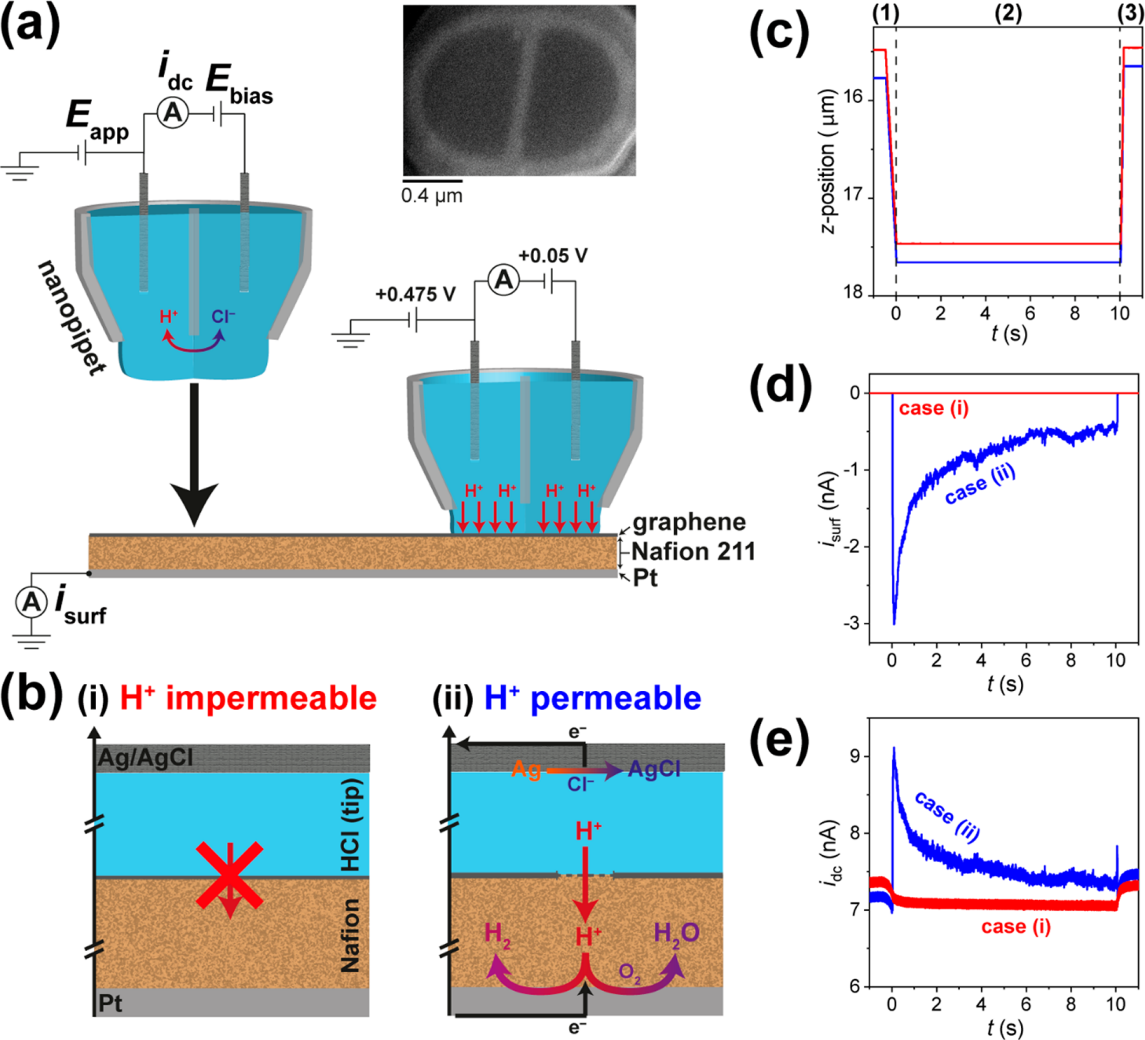
(a) Schematic of the SECCM set up employed herein. The dual-channel micropipet probe (representative SEM image shown, inset) is filled with electrolyte solution (*e.g.*, 0.1 M HCl) and equipped with identical Ag/AgCl QRCEs. During operation, *E*_bias_ is applied between the QRCEs, and the resulting *i*_dc_ is used as a feedback signal to detect meniscus–surface contact. A potential of *E*_app_ was applied to one of the QRCEs to control the Pt WE potential (*E*_surf_), where *E*_surf_ = −(*E*_app_ + *E*_bias_/2), and the WE surface current (*i*_surf_) was measured. (b) Schemes showing meniscus–surface contact with (i) proton-impermeable (red) and (ii) proton-permeable (blue) regions of the CVD graphene|Nafion membrane, along with representative plots of (c) *z*-position, (d) *i*_surf_, and (e) *i*_dc_. In case (i), ion flow between the WE and QRCEs is blocked by the impermeable graphene layer; no electrochemistry can occur at the Pt WE; *i*_surf_ is zero; *i*_dc_ only responds to making/breaking meniscus–surface contact. In case (ii), there is ion flow at proton-permeable sites of the graphene layer; proton-consuming reactions (HER and/or ORR) occur at the Pt WE; *i*_surf_ is nonzero; *i*_dc_ responds to making/breaking meniscus–surface contact and also reflects *i*_surf_ flowing at the WE (*i.e.*, the counter electrode current). The plots in (c–e) were obtained with *E*_bias_ = 0.05 V, *E*_app_ = 0.475 V, and *E*_surf_ = −0.5 V and are divided into three distinct stages, indicated by dashed lines in (c): (1) approach (*i.e.*, *t* < 0 s), (2) application of electrochemical waveform (*i.e.*, 0 ≤ *t* ≤ 10 s), and (3) retract (*i.e.*, *t* > 10 s). Note that protons are denoted as H^+^ in this figure.

Herein, an “electrochemical ion (proton) pump cell” configuration of SECCM is introduced to measure local proton transmission through graphene|Nafion membranes, as detailed in the Methods section and shown schematically in Figure 1a. During measurement, the dual-channeled micropipet probe (typical major and minor radii of ∼0.7 and 0.5 μm; herein, see Figure 1a inset) was filled with electrolyte solution (0.1 M HCl, unless otherwise stated). A bias voltage (*E*_bias_ in Figure 1a) was applied between the Ag/AgCl quasi-reference counter electrodes (QRCEs) located in the two channels, inducing an ion conductance current (*i*_dc_ in Figure 1a) to flow through the meniscus located at the end of the micropipet (referred to as the meniscus cell, hereafter). The *i*_dc_ is highly sensitive to deformation of the meniscus cell,^25,47^ meaning that it can be used to detect meniscus–surface contact, enabling accurate positioning of the SECCM probe in three-dimensional (3D) space.^48^

Electrical contact was made through a bottom contact of the Pt|Nafion|graphene electrode assembly, with meniscus top contact from the SECCM tip at the graphene overlayer. In the event where there is a path of ion flow between the Ag/AgCl QRCEs in the tip and the Pt working electrode (WE), through the graphene|Nafion membrane, the effective potential at the WE surface is *E*_surf_ = −(*E*_app_ + *E*_bias_/2) [*e.g.*, in Figure 1a, *E*_surf_ = −(0.475 + 0.05/2) V = −0.5 V *vs* Ag/AgCl_QRCE_].^25,47^ Depending on the value of *E*_surf_ (*vide infra*), two proton-consuming reactions can take place at the Pt WE, the hydrogen evolution reaction (HER) and/or the oxygen reduction reaction (ORR):
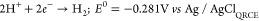
1
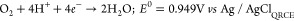
2At a sufficiently driving *E*_surf_, two scenarios are possible. Case (i): If the graphene film is ion-impermeable, the ionic pathway between the Pt WE and Ag/AgCl QRCEs is blocked and no electrochemistry can place at the Pt WE, as shown in Figure 1b-i. Case (ii): If the graphene film is ion-permeable, the electrochemical circuit is closed (*i.e.*, there is a continuous ionic pathway between the Pt WE and Ag/AgCl QRCEs) and protons flow from the meniscus cell into the Nafion film as the HER and/or ORR take place at the Pt WE (while the Ag/AgCl counter reaction takes place at the QRCE), as shown in Figure 1b-ii. Thus, the SECCM configuration shown in Figure 1a effectively represents an electrochemical ion (proton) pump cell,^40^ whereby protons are “pumped” across the graphene film in one direction (from meniscus to Nafion) in response to the proton-consuming reactions (eqs 1 and 2) at the Pt WE surface.

**Figure 2:**
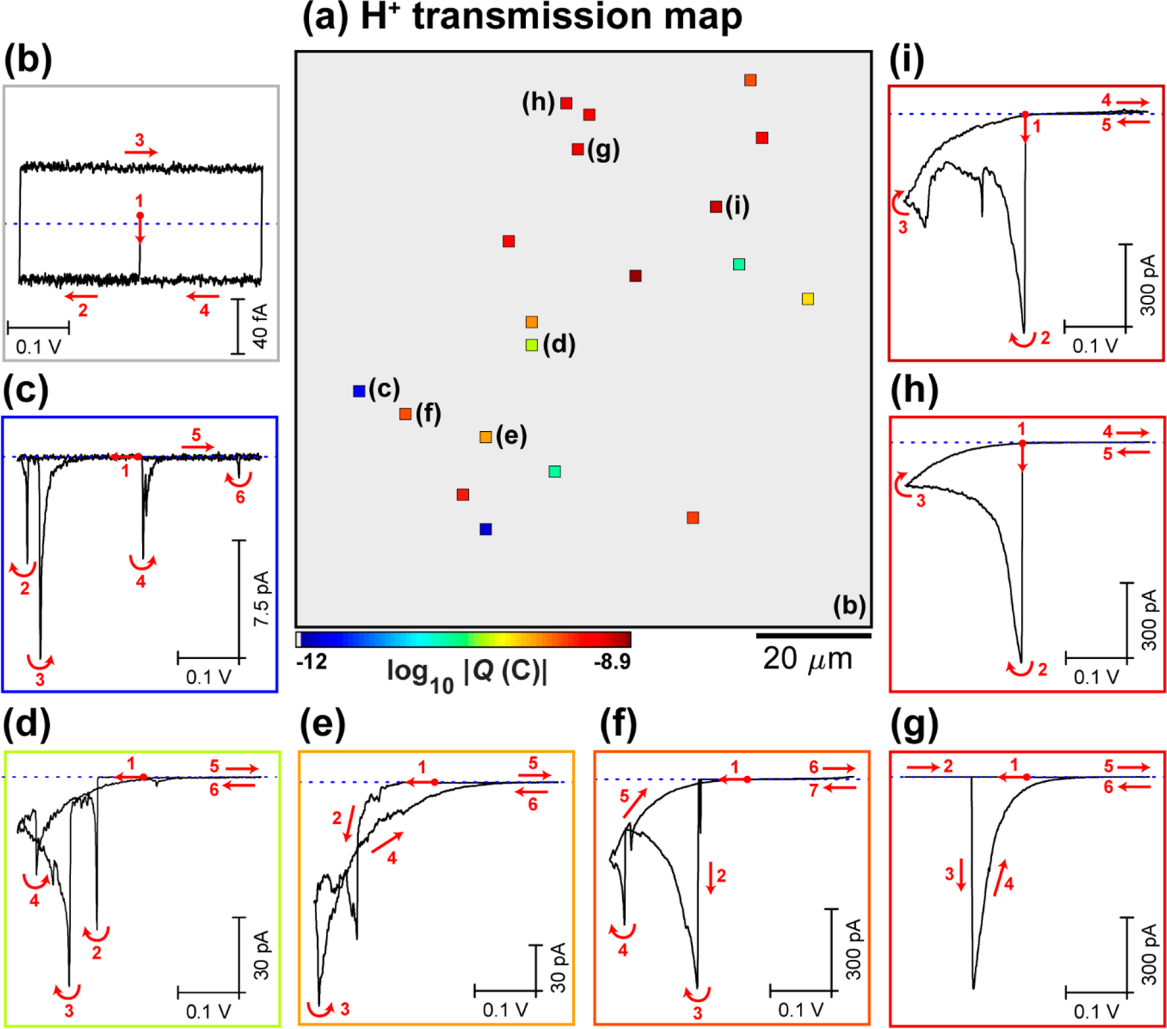
(a) Static image of electrochemical activity (proton transmission), collected over a 100 × 100 μm^2^ area of the graphene|Nafion membrane, using SECCM in the voltammetric (υ = 0.1 V s^–1^, 1 cycle) hopping mode configuration (hopping distance = 2 μm, 51 × 51 pixels, tip area ≈1 μm^2^). This image was obtained by integrating (with respect to time) the spatially resolved |*i*_surf_| data from SI Movie S1, over the entire *E*_surf_ range (−0.225 to 0.175 V *vs* Ag/AgCl_QRCE_). (b–i) Spatially resolved CVs (*i*_surf_–*E* curves), obtained by (b) averaging all inactive pixels (*N* = 2582) or (c–i) from the individual (representative) active pixels, indicated in (a). The border of each CV in (b–i) corresponds to the corresponding pixel colors in (a). Note that in (a), pixels with log_10_|*Q*| values less than 3× the electrical noise level are assigned a gray color and are considered to be inactive.

Plots of *z*-position, surface current (*i*_surf_), and *i*_dc_ from representative case (i) (red trace) and case (ii) (blue trace) measurements are shown in Figure 1c–e, respectively. The plots are divided into three distinct stages: (1) approach (*i.e.*, *t* < 0 s), (2) application of the electrochemical waveform (*i.e.*, constant potential at 0 ≤ *t* ≤ 10 s), and (3) retract (*i.e.*, *t* > 10 s). From the plot of *z*-position in Figure 1c, in both cases, the following sequence of events takes place: (1) the SECCM probe is translated toward the graphene|Nafion surface at a constant rate (4 μm s^–1^ in Figure 1c–e) until the *i*_dc_ set point is triggered (marked as *t* = 0 s in Figure 1c–e); (2) the probe position is held constant, as the electrochemical waveform is applied; and (3) the probe is retracted from the surface at a constant rate (15 μm s^–1^ in Figure 1c–e).

In reference to the plot of *i*_surf_ in Figure 1d, the following sequence of events takes place: (1) in both cases, *i*_surf_ is initially zero during approach, as meniscus–surface contact has not yet been established (*i.e.*, the electrochemical circuit has not been closed); (2) after establishing meniscus–surface contact, zero or nonzero *i*_surf_ is measured at the Pt WE at 0 ≤ *t* ≤ 10 s, indicating proton-impermeable [case (i)] and proton-permeable [case (ii)] regions of the graphene|Nafion membrane, respectively; (3) in both cases, *i*_surf_ returns to zero during retract, as meniscus–surface contact is broken. Note that the magnitude of *i*_surf_ is dependent on *E*_surf_ and may be limited by a combination of the charge-transfer resistance (*R*_ct_) associated with the electrode reaction (*i.e.*, HER and/or ORR at the Pt WE), the micropipet tip resistance (*R*_tip_), and the resistance of the proton transmission site(s) in the graphene film, explored in greater detail below.

From the plot of *i*_dc_ in Figure 1e, the following sequence of events takes place: (1) in both cases, *i*_dc_ initially adopts a constant value of ≈7 nA, which decreases during approach, until reaching the *i*_dc_ set point (feedback threshold; ±500 pA in Figure 1c–e); (2) in case (i), *i*_dc_ maintains a constant value, indicating a stable meniscus–surface contact, whereas in case (ii), *i*_dc_ increases dramatically at 0 ≤ *t* ≤ 10 s, reflecting a percentage of the counter current flowing between the Pt WE and QRCEs (≈56% of *i*_surf_ herein, with the other ≈44% being passed at the other QRCE); (3) in both cases, *i*_dc_ tends back toward a stable value during retract (similar to the value during approach), as the meniscus comes away from the surface. Note that the *i*_dc_–distance characteristic during the approach and retract of the SECCM tip depends on the tip size, initial meniscus size in air (here relatively large), and nature of the meniscus–substrate interaction (here, relatively small). The gradual decrease in *i*_dc_ on approach prior to triggering the feedback threshold at *t* = 0 s indicates substantial “squashing” of the meniscus cell^47^ over a distance of ≈500 nm in Figure 1c–e (*i.e.*, on the order of the micropipet probe radius). This *i*_dc_–distance behavior is similar to that observed previously for SECCM on suspended graphene, where meniscus contact to the graphene surface was also evident from direct simultaneous measurements of *i*_surf_.^49^ Here, meniscus contact was additionally confirmed in separate measurements of *i*_surf_ for the direct electrochemistry of FcDM^0/+^ at the graphene|Nafion substrate (*vide supra*). It should also be noted that, herein, the *i*_dc_ set point (±500 pA) is taken relative to the value measured at the beginning of the approach, which means that it is both insensitive to drift in *i*_dc_ (*i.e.*, self-referencing feedback^48^) and can be triggered by either a decrease [*i.e.*, during meniscus squashing, case (i)] or increase [*i.e.*, when the counter current flows due to proton transmission, case (ii)] in the magnitude of *i*_dc_, therefore serving as a sensitive indicator of meniscus–surface contact, irrespective of proton transmission.

### Local Proton Transport Dynamics through Graphene|Nafion Membranes

Potential- and time-dependent proton transmission through graphene|Nafion membranes was investigated locally using SECCM in the voltammetric hopping mode.^24,50^ A spatially resolved electrochemical movie, comprising 2601 (*i.e.*, 51 × 51 pixels) independent cyclic voltammograms (CVs) across an 100 × 100 μm^2^ area (hopping distance = 2 μm) in the potential range −0.225 to +0.175 V *vs* Ag/AgCl_QRCE_ (voltammetric scan rate, υ = 0.1 V s^–1^) is shown in the SI Movie S1 (associated movie caption presented in the SI). A colocated “quasi-topographical” map, which reflects the (dynamic) topology of the underlying Nafion membrane (*i.e.*, the atomically thin graphene layer conforms to the physical structure of the Nafion) collected synchronously with the electrochemical data, is presented in the SI Figure S3. Note that the ORR (eq 2) is the only reaction possible at the Pt WE within this potential range; that is, O_2_ serves as the depolarizer at the Pt WE surface. The corresponding static image of electrochemical activity (*i.e.*, proton conductance), obtained by integrating |*i*_surf_| from SI Movie S1 to calculate the charge (|*Q*_surf_|) passed over the entire potential range (details in the Methods section), is shown in Figure 2a.

From Movie S1 and Figure 2a, it is immediately evident that proton transmission through graphene|Nafion membranes is highly localized, detected at only 19 out of 2601 pixels (sites). Taking the area probed by the SECCM meniscus cell (*i.e.*, droplet footprint) to be equal to the tip area (≈1 μm^2^ in Figure 2), this corresponds to a proton transmission site density of ≈0.007 sites/μm^2^ [≈active pixels/(total pixels × tip area)]. Note that the active pixels do not necessarily correspond to obvious features in the quasi-topographical map of the graphene|Nafion membrane (see SI Figure S3), and the *i*–*E* response is different from that of the Nafion film itself (*vide infra*). Thus, the data in Figure 2a demonstrate that proton transport occurs at specific rare sites (*e.g.*, defects,^15−18^*vide infra*) across the macroscopic graphene|Nafion membrane investigated herein. Indeed, due to the high sensitivity and low electronic noise of the SECCM setup, through-plane proton conduction at the “inactive” pixels (*N* = 2582) can be effectively ruled out in these devices, as the associated low *i*_surf_ values (±40 fA, Figure 2b) throughout the investigated potential range are attributable to stray capacitance.

As discussed above, hydrated Nafion is effectively an isotropic (homogeneous) proton source/sink (electrolyte) on the scale of the SECCM probe (≈micrometer scale; see SI Figure S2a). Considering the weight of statistics presented in Figure 2, we can state with confidence that there is no detectable isotropic through-plane proton conduction across the graphene|Nafion membrane. Proton transmission is highly localized and most likely a defect-driven process (*vide infra*), and in these locations, proton transmission rates (currents) are very high. However, as Nafion does not possess a uniform structure on the scale of (atomic) defects (*i.e.*, sub-nanoscale), the structure-dependent local proton conductivity of the acceptor Nafion membrane needs to be acknowledged. From the classical cluster-network (inverted micelle) model for the morphology of hydrated Nafion,^51^ proton conduction through the graphene|Nafion membrane could only occur if a proton-conducting defect site (∼nanometers to sub-nanometer scale) of graphene aligns with a proton-accepting water channel (∼nanometers to 10 nm scale) of Nafion (shown schematically in the SI Figure S2b). Given the high hydration state of the Nafion membrane (*e.g.*, proton conductive surface area of at least 50% at 70% relative humidity^44^), there would be an abundance of proton receptor sites on the 1–2 μm^2^ scale (*i.e.*, scale of the SECCM probe), evidenced by the high functionality of graphene|Nafion membranes in previous macroscopic studies.^11,12,19^ Thus, while we are confident that we are not simply measuring a sparsity of graphene|Nafion wetting in the proton receptor phase (sink) in our measurements in Figure 2, it is prudent to take the site densities measured herein (*e.g.*, 0.007 sites/μm^2^, *vide supra*) as a lower limit for these CVD graphene|Nafion membranes.

As shown in the SI Movie S1, in addition to being highly localized, proton transmission through the graphene|Nafion membrane in these measurements is also a highly dynamic process, with the number of active pixels and magnitude of *i*_surf_ varying from frame-to-frame. To demonstrate this more clearly, individual CVs, extracted from representative active pixels, are plotted in Figure 2c–i. In many cases, *i*_surf_ is initially at the sub-picoampere baseline (*e.g.*, see Figure 2b) before “spiking”, sometimes exhibiting multiple small transient events (*i.e.*, on the order of 1 pA to tens of pA, Figure 2c,d) and in other cases one or more large event(s) (*i.e.*, |*i*_surf_| > 100 pA, Figure 2e–g). This indicates that proton transmission sites may locally “open” and in some cases apparently “close” (*e.g.*, Figure 2c) as a function of potential and/or time, and that the dimensions of these sites (reflected by the magnitude of *i*_surf_, *vide infra*) may also vary. In other cases, *i*_surf_ is nonzero from the beginning of the potential sweep (*i.e.*, |*i*_surf_| > 100 pA at the starting potential, −0.025 V *vs* Ag/AgCl_QRCE_, Figure 2h,i). While the application of an electric field across monolayer membranes of graphene^52^ and other 2D materials^53^ can nucleate nanopores that facilitate local ion transfer, this is typically achieved using voltage pulses that are ultrashort and high intensity (*e.g.*, 7 V for 250 ns)^4,52^ relative to those employed herein. It should be noted, however, that during ultrashort/high-intensity voltage pulses, the actual magnitude of the potential/electric field over the graphene membrane is expected to be dramatically reduced due to double layer charging and uncompensated resistance, which, as discussed below, can be avoided entirely through the application of low-intensity voltage pulses for long times (*e.g.*, ≤ 0.5 V for >1 s).

In each of the CVs extracted from active pixels (Figure 2c–i), individual *i*_surf_ spikes are always followed by a relatively slow exponential decay with potential/time, taking place on the millisecond to second time scale (see Figure 2g). As alluded to above, this slow decay is associated with the charging of electrical double layer(s) [*i.e.*, double layer capacitance (*C*_dl_) of the macroscopic Pt WE], through the uncompensated resistance of the cell, the time scale of which is characterized by the *RC* time constant (τ).^54^ As discussed in the next section (and outlined in detail in the SI), over an active proton transport site, *R* and *C* are estimated to be on the order of ≈100–1000 MΩ and ≈2 nF, respectively, giving rise to τ values of 0.2–2 s, consistent with the time scale of the decay in *i*_surf_. In addition, in Figure 2c–i, *i*_surf_ is negative (*i.e.*, corresponding to a reduction process at the Pt WE) and shows an exponential dependence on (over)potential, starting at ≈0 V *vs* Ag/AgCl_QRCE_. This indicates that in the potential range of 0 to −0.225 V *vs* Ag/AgCl_QRCE_, both the relatively sluggish ORR kinetics at the Pt WE surface and the geometry of the active transmission site may contribute some limitation to the magnitude of *i*_surf_ (and hence the total reactive flux of protons across the graphene|Nafion membrane). These points are further discussed below.

**Figure 3:**
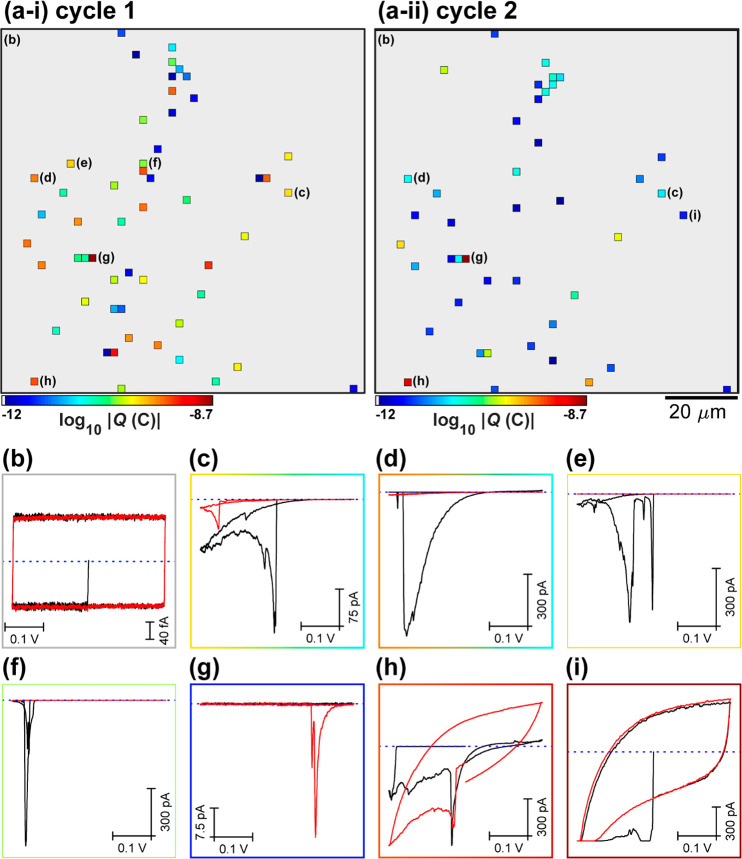
(a) Static images of electrochemical activity (proton transmission), collected over a 100 × 100 μm^2^ area of the graphene|Nafion membrane, using SECCM in the voltammetric (υ = 0.2 V s^–1^, 2 cycles) hopping mode configuration (hopping distance = 2 μm, 51 × 51 pixels, tip area ≈1 μm^2^). (i) Cycle 1 and (ii) cycle 2 are shown separately. These images were obtained by integrating the spatially resolved |*i*_surf_| data from SI Movie S2 over the entire *E*_surf_ range (−0.225 to 0.175 V *vs* Ag/AgCl_QRCE_). (b–i) Spatially resolved CVs (*i*_surf_–*E* curves), obtained by (b) averaging all inactive pixels (*N* = 2544) or (c–i) from the individual (representative) active pixels, indicated in (a). Cycles 1 and 2 are represented by the black and red traces, respectively.

To explore the dynamics of proton transport, particularly the potential dependence, a voltammetric hopping mode SECCM experiment was performed on another area of the graphene|Nafion membrane, performing two cycles within the same potential window (−0.225 to +0.175 V *vs* Ag/AgCl_QRCE_). A spatially resolved electrochemical movie, comprising 2601 independent CVs (51 × 51 pixels, υ = 0.2 V s^–1^) across an 100 × 100 μm^2^ area (hopping distance = 2 μm) is shown in the SI Movie S2. The corresponding static images of electrochemical activity (*i.e.*, proton conductance) obtained for cycles 1 and 2 are shown in Figure 3a-i and a-ii, respectively (synchronously obtained quasi-topography map presented in the SI Figure S4). By consulting Movie S2 and Figure 3a, it is again clear that proton transmission through graphene|Nafion membranes is a highly localized and dynamic phenomenon, occurring at 57/2601 pixels (sites), corresponding to ≈0.02 sites/μm^2^. While a large proportion of the proton transmission sites are fixed, there are some sites that “open” and others that (partly) “close” on the time scale of the measurement (*i.e.*, compare Figure 3a-i and a-ii). This apparent “opening” and “closing” may be due to changes in the transmission site in the graphene itself (*e.g.*, structural fluctuations induced by changes in local charge or adsorption of impurities;^4^ transient wetting/dewetting^55^ or nanobubble nucleation^56^ in/at the transmission site) or dynamics of the acceptor Nafion phase^57^ (see SI Figure S2b). These are further reasons to consider the density of transmission sites that we report as a lower limit. In any case, most of the graphene|Nafion membrane is impermeable to protons, clearly demonstrated in the average CVs (*N* = 2544) shown in Figure 3b (note that the current from stray capacitance scales with υ and is therefore double that shown in Figure 2b).

Individual CVs extracted from representative active pixels are plotted in Figure 3c–i. Consistent with Figure 2c–i, the |*i*_surf_| “spikes” either upon meniscus landing (Figure 3c) or after sweeping the potential (Figure 3d–g) and is followed by an exponential decay on the millisecond to second time scale. This decay may explain why *i*_surf_ is typically lower on the second voltammetric cycle compared to the first (*e.g.*, Figure 3c–f), although in some cases, the opposite is true (*e.g.*, Figure 3i). In a select few pixels (4/2601, Figure 3a), very large *i*_surf_ values are measured during meniscus–surface contact, giving CVs that exhibit very large capacitive current envelopes, as demonstrated in Figure 3h,i. In these instances, the meniscus cell has landed directly on Nafion that has extruded through the graphene layer, proven by comparison to the response when performing an SECCM scan on a relatively defective area of the graphene|Nafion membrane (see SI Figure S5 and associated discussion).

### Estimating the Dimensions of the Proton-Conducting Sites

**Figure 4:**
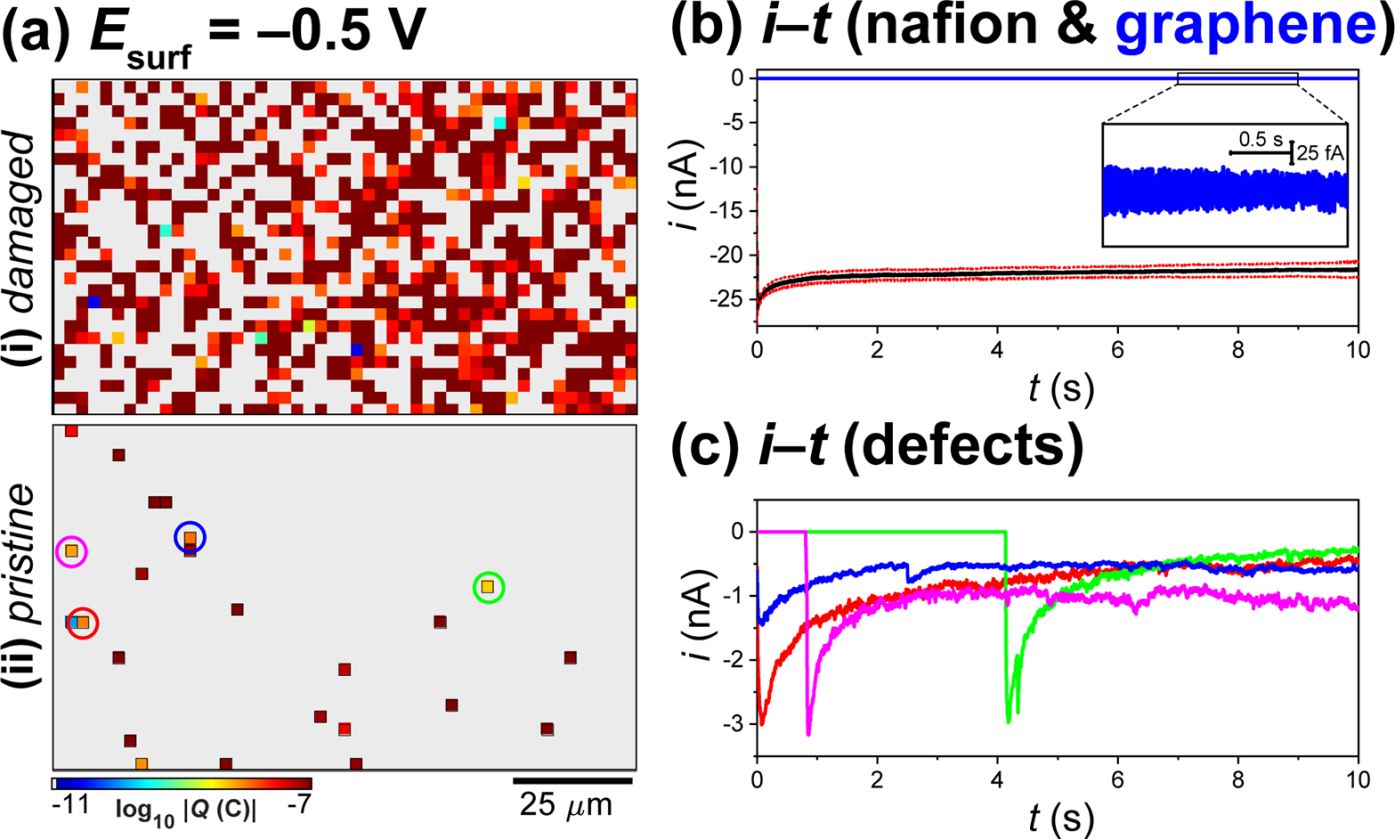
(a) Static images of electrochemical activity (proton transmission), collected over a 120 × 70 μm^2^ area of a graphene|Nafion membrane, using SECCM in the amperometric (*E*_surf_ = −0.5 V *vs* Ag/AgCl_QRCE_, *t* = 10 s) hopping mode configuration (hopping distance = 2.5 μm, 49 × 29 pixels, tip area ≈2 μm^2^). (i) “Damaged” and (ii) more intact (“pristine”) areas of the graphene|Nafion membrane are shown. These images were obtained by integrating the spatially resolved |*i*_surf_| data from SI Movies S3 and S4. (b) Chronoamperograms (*i*_surf_–*t* curves) extracted from (a-i), obtained from areas of the membrane where the Nafion is extruded through the graphene overlayer [*i.e.*, dark-red pixels in (a-i); black trace in (b)] and the graphene overlayer remains intact [*i.e.*, gray areas in (a-i), blue trace in (b)]. The Nafion curve in (b) was obtained by selecting 10 off-scale (*i.e.*, dark-red) pixels that are surrounded by active pixels in (a); the resulting average (black line) ± one standard deviation (red dashed lines) curves are shown. (c) Chronoamperograms extracted from the individual proton transmission sites (pixels) labeled in (a-ii).

Proton conduction through local transmission sites on the graphene|Nafion membranes is likened to ion transport through an atomically thin, solid-state nanopore.^4,41^ Applying the equivalent circuit model^58,59^ derived and discussed in the SI, the local electrochemical response (*e.g.*, *i*–*E* or *i*–*t*) is rationalized and further used to estimate the geometry of the active transmission sites. To achieve the latter, potential-step (*i.e.*, chronoamperometry) experiments were performed in the SECCM configuration, targeting both damaged and more intact areas of the graphene|Nafion membrane. Spatially resolved electrochemical movies, comprising 1421 independent chronoamperograms (CAs) across 120 × 70 μm^2^ areas (hopping distance = 2.5 μm, 49 × 29 pixels) at *E*_surf_ = −0.5 V *vs* Ag/AgCl_QRCE_ (pulse time = 10 s) are shown in the SI Movies S3 and S4. The corresponding static images of electrochemical activity (*i.e.*, proton transmission), obtained from damaged and more intact areas of the graphene|Nafion membrane, are shown in Figure 4a-i and a-ii, respectively.

Contrary to the SECCM scans shown above (Figures 2 and 3), in the damaged area of the graphene|Nafion membrane (Figure 4a-i), there are large regions of grouped active pixels (704/1421), separated by areas of inactive pixels (717/1421). Notably, the inactive pixels, corresponding to areas of intact graphene film, remain impermeable to protons, evident from the average *i*–*t* curve shown in Figure 4b. Conversely, at the active pixels, which mostly correspond to macroscopic defects (*e.g.*, cracks and holes) where the underlying Nafion extrudes through the graphene overlayer, relatively large *i*_surf_ values of ≈−23 nA are measured throughout the entire *i*–*t* pulse, also shown in Figure 4b. In contrast to the damaged area (Figure 4a-i), the more intact area of the graphene|Nafion membrane (Figure 4a-ii) resembles the previous SECCM scans (Figures 2 and 3), with only a small number of active pixels (*N* = 24/1421), surrounded by contiguous areas of intact, proton-impermeable graphene. While many of the active pixels exhibit large *i*_surf_ values (*i.e.*, the dark red pixels in Figure 4a-ii), comparable to that obtained from the Nafion itself (*e.g.*, black trace, Figure 4b), some others pass much smaller currents, as demonstrated in Figure 4c. At these sites, *i*_surf_ spikes either at the beginning of the *E*–*t* pulse (*e.g.*, blue and red traces) or after an onset time (*e.g.*, ≈1 and ≈4 s for the pink and green trace, respectively), before decaying on the millisecond to second time scale (consistent with τ ≈ 0.2–2 s, discussed in the SI) to steady values in the 270–1100 pA range. Again, the pixel-dependent delayed onset of the *i*_surf_ spike in Figure 4c serves to highlight that proton transmission through graphene|Nafion membranes shows strong time dependence (Movies S3 and S4).

The large driving potential of −0.5 V *vs* Ag/AgCl_QRCE_ (η ≈ 0.2 V, discussed in the SI) and long pulse time of 10 s applied during these potential-step experiments permits quantitative treatment of the data, allowing the pore resistance (*R*_pore_) to be calculated and the pore radii (*r*_p_) to be estimated. As seen from SI Figure S6, assuming *C*_dl_ and *R*_ct_ can be neglected, and under conditions where *R*_pore_ is negligible (*i.e.*, by landing directly on the extruded Nafion film itself), the series resistance (*R*_series_) ≈ *R*_tip_, meaning that *i*_surf_ is limited by the resistance of the micropipet probe. Applying Ohm’s law (SI eq S13), *R*_series_ is estimated to be ≈9 MΩ from *i*_surf_ = −23 nA (Figure 4b), which is consistent with *R*_tip_, estimated to 6–8 MΩ from *i*_dc_ = 7 ± 1 nA (SI eq S7). Thus, landing on the Nafion film provides the “tip-limited” *i*–*t* response, and pixels with *i*_surf_ values approaching approximately −23 nA (*i.e.*, the dark red pixels in Figure 4a-ii) are precluded from further quantitative treatment (*i.e.*, if *R*_pore_ ≪ *R*_tip_ then *R*_series_ ≈ *R*_tip_).

Since *R*_tip_ is known (≈9 MΩ), *R*_pore_ can be calculated (SI eq S14) for each of the individual active pixels highlighted in Figure 4a-ii,c. *R*_pore_ values of 730, 460, 320, and 170 MΩ are calculated for *i*_surf_ values of 270, 430, 600, and 1100 pA, for the green-, red-, blue-, and pink-labeled pixels (Figure 4a-ii), respectively. Assuming ρ = 25 Ω·cm (calculated from κ = 0.04 S cm^–1^ for 0.1 M HCl^60^) and taking *L*_p_ = 0.6 nm (the apparent thickness of graphene in water^7^), we estimated *r*_p_ values of 0.4, 0.5, 0.6, and 1.0 nm (SI eq S12) for the green-, red-, blue-, and pink-labeled pixels (Figure 4a-ii), respectively. As discussed in the SI, in a regime where pore radius and pore length are similar (*i.e.*, *r*_p_ ≈ *L*_p_), both the access resistance (*R*_g_ ∝ 1/*r*_p_) and geometric resistance (*R*_a_ ∝ *L*_p_/r_p_^2^) contribute significantly to *R*_pore_, and the calculated *r*_p_ values are sensitive to *L*_p_ (SI eq S12). For instance, taking *R*_pore_ = 460 MΩ, *r*_p_ is estimated to be 0.4 and 0.6 nm for *L*_p_ values of 0.34 nm (*i.e.*, van der Waals diameter of carbon atoms) and 1 nm (*i.e.*, the upper limit of reported values for the apparent thickness of graphene in water^4^), respectively. In any case, the estimated (sub)nanometer pore geometry from this simple model indicates that the local proton transmission sites through the macroscopic graphene|Nafion membrane likely coincide with relatively rare atomic-scale defects (naturally occurring or introduced, *vide infra*) in the graphene overlayer film, consistent with some previous reports.^15−18^

A summary of example *G*/*A* values reported in previous studies, alongside the graphene preparation method and size of the measured membrane, is reported in the SI Table S1. The *G/A* values previously reported for macroscopic graphene|Nafion membranes varies over several orders of magnitude (≈0.09 to 30 S cm^–2^),^11,12,19^ which is perhaps unsurprising given that macroscopic defects such as pinholes, cracks, and other imperfections are known to be present. Indeed, comparing Figure 4a-i and a-ii, it is clear that the quality of the graphene overlayer can be highly variable within a given graphene|Nafion membrane.

To contextualize the results reported herein, the density of defects (defects μm^–2^) required to achieve the reported *G*/*A* values is also calculated, assuming an individual defect resistance of 170 MΩ·defect. As shown in Table S1, the lower end of defect densities (0.005 defects μm^–2^), obtained from high-quality, small-area graphene membranes produced by exfoliation (3 mS cm^–2^, reported^5^) or CVD (4 mS cm^–2^, reported^18^), is in good agreement with the number of defects detected on the more pristine areas of the graphene|Nafion membrane, with values of 0.007, 0.02, and 0.008 defects μm^–2^ calculated for Figure 2a, Figure 3a, and Figure 4a-ii (assuming 1 defect/pixel), respectively. To match the highest-performing defect-engineered graphene membranes (*G*/*A* values of up to ≈1000 mS cm^–2^),^17^ the density of defects would need to increase by >2 orders of magnitude (assuming a constant defect resistance of 170 MΩ·defect) up to ∼2 μm^–2^, such that on average each meniscus cell of size ≈2 μm^2^ (Figure 4) would contain four defects. While additional proton transmission sites appear *in situ* (*e.g.*, see Movie S4), previous reports have shown that such defects can be introduced readily during the growth^17^ or postgrowth treatment (*e.g.*, plasma etching^18^) of CVD graphene, producing highly conductive, proton-selective membranes.

### High-Resolution Imaging

**Figure 5:**
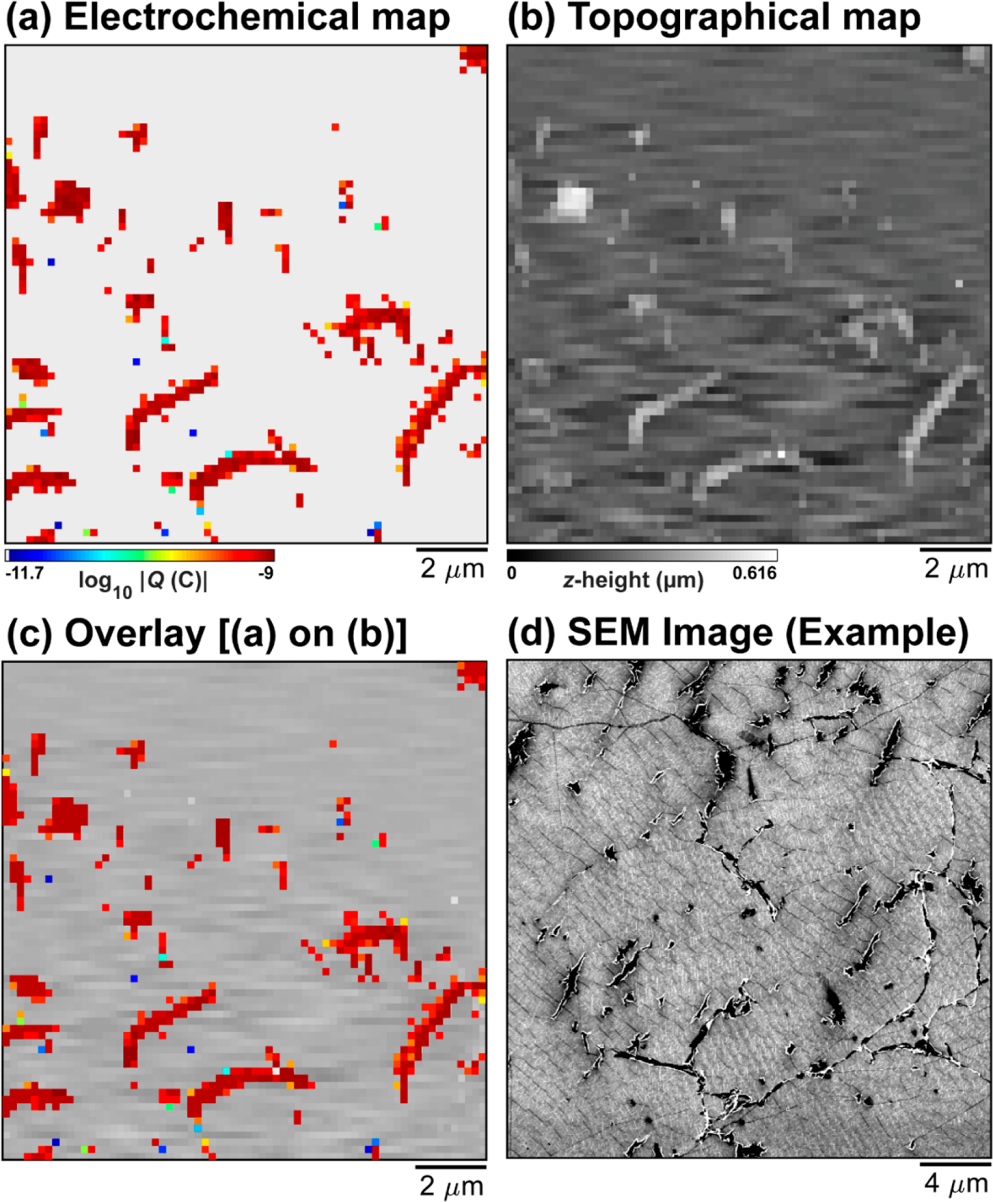
(a) High-resolution electrochemical activity (log_10_|*Q*|) and (b) colocated quasi-topographical maps (measured synchronously), collected over a 13.4 × 13.8 μm^2^ area of a graphene|Nafion membrane, using SECCM in the voltammetric (υ = 1 V s^–1^, 1 cycle, *E*_surf_ = ± 0.25 V *vs* Ag/AgCl_QRCE_) hopping mode configuration (hopping distance = 200 nm, 68 × 70 pixels, tip area ≈ 0.004 μm^2^). (c) Overlay of (a) on (b). (d) SEM image obtained from an adjacent area of the graphene|Nafion membrane, exhibiting similar features (*i.e.*, macroscopic defects such as cracks and pinholes) as those imaged in (a) and (b).

To provide a closer inspection of graphene|Nafion membranes, a much smaller nanopipet probe (*r*_t_ ≈ 30–40 nm, image shown in the SI Figure S7) was employed to target a relatively defective area of the membrane. A static map of electrochemical activity made up of 4760 pixels across an 13.4 × 13.8 μm^2^ area (hopping distance = 200 nm, 68 × 70 pixels) is shown in Figure 5a. Evidently, while a majority (4286/4760 pixels) of the graphene|Nafion membrane remains inactive, the finer probe reveals detail that was previously not seen with the larger probes (*r*_t_ ≈ 0.6–1 μm), with a small number of isolated (single-pixel) defects possessing low activity (*i.e.*, blue pixels) and a large number of continuous (multipixel) defects possessing high activity (*i.e.*, dark red pixels). The synchronously collected, colocated quasi-topography map shown in Figure 5b reveals that the single-pixel defects do not coincide with topographical defects, in agreement with the measurements performed above (*e.g.*, Figure 2), whereas the multipixel ones coincide with areas of elevated topography. Overlaying Figure 5a on Figure 5b demonstrates this more clearly, as shown in Figure 5c. On this basis, it is concluded that the single-pixel sites likely coincide with the atomic-scale defects that accommodate selective proton transmission (*vide supra*), whereas the larger multipixel sites represent areas where the underlying Nafion film has extruded through the graphene overlayer, most likely at pre-existing cracks or grain boundaries. Indeed, macroscopic defects of this type can also be observed by scanning electron microscopy (SEM) imaging, carried out on a nearby area of the graphene|Nafion membrane, as shown in Figure 5d.

It should be pointed out that while a small SECCM probe provides high-resolution images, a relatively large probe (*r*_t_ ≈ 0.6–1 μm, Figure 1a, inset) is advantageous for quantitative measurements. This is because the scan area generally scales with the probe size, meaning that when the density of transport sites is low (0.007–0.02 μm^–2^, *vide supra*), relatively large areas of membrane can be covered in a single SECCM scan. In addition, to accurately estimate *R*_pore_ from *R*_series_ (SI eq S13), *R*_tip_ ≪ *R*_pore_, which puts a lower limit on the probe size since *R*_tip_ ∝ 1/*r*_tip_ (eq S6).

## Conclusions

In this study, an “electrochemical ion (proton) pump cell” configuration of SECCM has been used to probe the spatially dependent proton permeability of CVD graphene|Nafion membranes. Due to the sheer weight of statistics (>5000 individual measurements, total, effectively corresponding to >5000 separate ion conductance devices) over several large areas (≈0.01 mm^2^) of the membrane, it can be stated with confidence that the majority of the graphene overlayer does not conduct protons in the investigated CVD graphene|Nafion membrane devices. Proton transmission was shown to be a site-specific process, occurring at ∼0.007–0.02 sites μm^–2^, giving rise to very high local conductance values (order of ≈1 S cm^–2^, normalized to the ∼1–2 μm^2^ footprint of the SECCM meniscus cell). In addition, proton transmission was strongly potential- and time-dependent, with additional transmission sites dynamically “opening” and a small number shutting off during the measurements. Reasons for this behavior have been suggested. A simple equivalent circuit model was proposed, analogizing these transmission sites to electrolyte-filled circular nanopores in the graphene film, which were estimated to possess dimensions (radii) on the (sub)nanometer scale, implying that atomic defects are responsible for local proton transport, in agreement with recent modeling^16^ and experimental^15,18^ works. The potentiality of SECCM for rapidly assessing the quality of ion-selective membranes was further demonstrated by deploying a fine nanopipet probe, producing high-throughput, high-resolution electrochemical and (quasi-)topographical images that gave a more detailed picture of the local proton transmission sites.

Overall, the results presented herein demonstrate the strong potential of SECCM as a multifunctional membrane characterization tool, producing high-fidelity images that provide a wealth of information on spatially resolved ion-selective transport/transmission. Although CVD graphene|Nafion membranes have been exclusively considered herein, ion-selective transport through membranes plays an important role in many applications, to name a few: electrochemical energy storage (*e.g.*, batteries) and conversion (*e.g.*, fuel cells, *vide supra*); separation technologies; and biological systems. Beyond membranes, SECCM may also have application in any areas where ion transport and/or reactive flux is a highly localized phenomenon, for example, in the characterization of corrosion-resistant coatings. Graphene has been proposed as a corrosion-resistant coating,^61^ and based on the results presented herein, it is clear that high-resolution, dual-channel SECCM could be deployed to rapidly assess local protection efficiency, in particular, by identifying activity “hot-spots” where the protective barrier may be compromised.

## Methods

### Chemical Reagents and Electrode Materials

Hydrochloric acid (HCl, 37%, Sigma-Aldrich), 1,1′-ferrocenedimethanol (FcDM, 97%, Sigma-Aldrich), and potassium chloride (KCl, 99.5%, Honeywell, Germany) were used as supplied by the manufacturer. All aqueous solutions were prepared with ultrapure deionized water (resistivity = 18.2 MΩ·cm at 25 °C, Integra HP, Purite, U.K.).

The Nafion 211 membranes were purchased from the Fuel Cell Store (College Station, TX). The monolayer CVD graphene (supported on copper foil) was purchased from ACS Material (Pasadena, CA). In previous studies, Raman spectroscopy indicated that these graphene-on-copper substrates (and graphene|Nafion membranes, *vide infra*) are high-quality, with no detectable D-peak near 1350 cm^–1^, indicating a relative lack of graphene defects with edge-plane character.^11,19^ Further characterization with X-ray photoelectron spectroscopy (XPS) revealed successful graphene transfer onto the Nafion membrane, with no detectable impurities or surface contamination from copper (see SI Figure S8). The XPS survey reveals the expected elemental composition (*i.e.*, F 1s, O 1s, C 1s, and S 2p) for both unmodified Nafion 211 and graphene|Nafion membranes (see SI Table S2). The C 1s spectra of an unmodified Nafion 211 membrane show only one major main carbon peak at 291 eV, attributed to the CF_2_ group of the fluorocarbon backbone (the other low intensity peak at 284.8 eV is assigned to adventitious carbon impurities). In contrast, C 1s spectra from the graphene|Nafion membranes show two main peaks, corresponding to the CF_2_ groups of Nafion and sp^2^ carbon atoms of graphene at 291 and 284.1 eV, respectively.

The nanocrystalline Pt WE was prepared by evaporating a 2 nm Cr adhesion layer followed by a 75 nm Pt layer on a borosilicate glass microscope slide. The glassy carbon plate was purchased from HTW Germany and was polished with a suspension of 0.05 μm Al_2_O_3_ (Buehler, Lake Bluff, IL), prior to use as a WE. Ag/AgCl QRCEs were prepared by anodizing 125 μm diameter Ag wire (99.99%, Goodfellow, U.K.) in an aqueous saturated KCl solution. The Ag/AgCl QRCEs possessed a stable reference potential (measured *vs* a commercial saturated calomel electrode, SCE) on the hours time scale in 0.1 M HCl, consistent with a previous report.^62^

### Pt|Nafion|Graphene Electrode Assembly Preparation

Nafion|graphene sandwich structures were fabricated at Clemson University, U.S.A., using a previously reported procedure.^11,19^ In brief, a ≈2 × 2 cm^2^ square of copper-supported graphene was placed on top of a Nafion 211 disk with a diameter of ≈1.9 cm and a thickness of ≈25 μm. Furthermore, two pieces of Teflon-reinforced fiberglass (of diameter ≈1.9 cm) were placed below and atop the Nafion|graphene on copper to serve as protective layers. This assembly was then placed into a hot press (Carver, Wabash, IN) and pressed at 140 °C for 2 min. Next, the Nafion|graphene|copper assembly was placed into a 0.3 M ammonium persulfate solution and allowed to react until the copper layer was fully etched away by visual inspection. Note that in previous studies,^11,19^ monolayer graphene sheets were shown to survive the Nafion hot-pressing and copper-etching processes intact, without any significant creation of additional defects or contamination from the copper substrate, as revealed by Raman spectroscopy and XPS.

Prior to scanning with SECCM, the graphene|Nafion assembly was rinsed in deionized water and fixed to a 2 × 2 cm^2^ nanocrystalline Pt WE with adhesive tape, ensuring intimate contact between the Nafion and Pt. The constructed Pt|Nafion|graphene electrode assembly was then fitted into a custom sample holder with a surrounding moat of deionized water,^38,47^ effectively fixing the local relative humidity at >70%. Due to the reportedly long equilibration times associated with Nafion hydration,^45,63^ the electrode assembly was stored under these conditions overnight. Following this procedure ensured that, during SECCM experiments on the several hours time scale, the prehydrated Nafion 211 membrane (1) possessed high bulk proton conductivity (on the order of 0.02–0.06 S cm^–1^)^42^ and (2) did not undergo significant changes in volume (*i.e.*, swelling/contraction). An electrical connection was made by fixing a copper wire to the Pt WE surface with conductive silver epoxy resin (RS Components, U.K.), taking care to avoid making a connection (*i.e.*, electrical short circuit) with the graphene overlayer film. Scanning electron microscopy imaging was carried out on the Pt|Nafion|graphene electrode assembly, after SECCM, with a GeminiSEM 500 system (Zeiss, Germany).

### Probe Fabrication

Double-barreled pipet probes, with total tip areas (*i.e.*, calculated from the overall diameter of the dual barrel) in the ≈10^–11^ to ≈10^–9^ cm^2^ (nanopipets) and ≈10^–8^ cm^2^ (micropipets) ranges, were fabricated from filamented quartz and borosilicate (Harvard Apparatus, Holliston, MA) theta capillaries, respectively, using a CO_2_ laser puller (P-2000, Sutter Instruments, Novato, CA). After fabrication, both barrels of the probes were backfilled with analyte solution (*e.g.*, 0.1 M HCl) using a MicroFil syringe (World Precision Instruments Inc., Sarasota County, FL), before adding a thin layer of silicone oil (DC 200, Sigma-Aldrich) on top to minimize evaporation from the back of the pipet during prolonged scanning, as previously reported.^33^ Ag/AgCl QRCEs were then inserted into each barrel, through the silicone oil layer, into the analyte solution, to finalize the SECCM probe, rendering it ready for use. After being scanned, the SECCM probes were carefully emptied and rinsed with deionized water (using a clean MicroFil syringe) before imaging the tip on a GeminiSEM 500 system.

### Scanning Electrochemical Cell Microscopy

Local electrochemical measurements were carried out in the SECCM format on a home-built scanning electrochemical probe microscopy (SEPM) workstation at the University of Warwick, U.K., as previously reported.^24,25,27,47^ In this configuration, the constructed SECCM probe (*i.e.*, filled theta-pipet equipped with QRCEs, *vide supra*) was mounted on a *z*-piezoelectric positioner (38 μm range, P-753.3, Physik Instrumente, Germany), and the Pt|Nafion|graphene electrode assembly (loaded in sample holder, *vide supra*) was mounted atop an *xy*-piezoelectric positioner (250 × 250 μm^2^ range, P-622.2, Physik Instrumente). As schematized in Figure 1a, a bias potential (*E*_bias_) of 0.05 V was applied between the QRCEs to induce a dc ion current (*i*_dc_) between the barrels to enable meniscus positioning on the substrate.^48^ The SECCM probe was initially positioned above the WE using coarse *xy*-micropositioners (M-461-XYZ-M, Newport, Irvine, CA) and subsequently lowered into the near-surface position using a stepper motor in tandem with an optical camera (PL-B776U, PixeLINK, Canada).

The SECCM probe (total tip area ≈10^–8^ cm^2^) was approached to the graphene overlayer film (*i.e.*, located at the *top* of the Pt|Nafion|graphene electrode assembly) surface using an *i*_dc_ threshold of ∼500 pA to detect when the meniscus–surface contact had been made and to stop further translation. Note that the glass portion of the probe never contacted the graphene surface. Electrochemical measurements (cyclic voltammetry or chronoamperometry, herein) were performed in the confined area defined by the meniscus cell created between the SECCM probe tip and graphene surface (*e.g.*, Figure 1a). During cyclic voltammetry, the potential at the Pt WE (*i.e.*, located at the *bottom* of the Pt|Nafion|graphene electrode assembly, Figure 1) was cycled between −0.225 and +0.175 V *vs* Ag/AgCl_QRCE_ (0.1 M Cl^–^) at voltammetric scan rates (υ) of 0.1 or 0.2 V s^–1^ for 1 or 2 cycles, respectively. During chronoamperometry, the potential at the Pt WE was held at −0.5 V *vs* Ag/AgCl_QRCE_ (0.1 M Cl^–^) for 10 s. Mapping was carried out using a standard hopping mode protocol, as previously reported.^29,50^ In brief, the SECCM probe was approached to the graphene surface at a series of locations in a predefined grid pattern, and upon each landing, an independent electrochemical measurement was made, building up spatially resolved chronoamperometric (*i*–*t*) or voltammetric (*i*–*E*) “images” of the substrate surface. In addition, the final position of the *z*-piezoelectric positioner at approach was used to synchronously construct a “quasi-topographical” map of the Pt|Nafion|graphene electrode assembly surface. Note that, in context, “quasi” refers to the fact that the underlying Nafion membrane possesses a dynamic physical structure (topology) due to small changes in volume (*e.g.*, contract/expansion in response to the humidity level) on the time scale of SECCM scanning (*vide supra*).

The SEPM setup was located on a vibration isolation platform (25BM-8, Minus K, Inglewood, CA) located within an aluminum faraday cage equipped with heat sinks and acoustic foam to minimize mechanical vibration, electrical noise, and thermal drift (<10 nm per minute) during prolonged scanning.^28,64^ The QRCE potentials were controlled, with respect to ground, with a home-built bipotentiostat, and the current flowing at the Pt WE (*i.e.*, surface current, *i*_surf_), held at a common ground, was measured with a home-built electrometer. Note that during the SECCM measurements, unless otherwise stated (*e.g.*, for the surface redox measurements used to assess the surface state, see SI Figure S1), the graphene membrane itself was floating (*i.e.*, it was neither biased nor electrically grounded). The *i*_surf_ and *i*_dc_ were measured every 4 μs and averaged in 256 blocks to give an effective data acquisition rate of 4 × (256 + 1) = 1028 μs, where one extra iteration was used to transfer the data to the host computer. A home-built eighth-order (low-pass) brick-wall filter unit (time constant = 1–10 ms) was utilized during data (current) collection. Instrumental control and data acquisition were carried out using an FPGA card (PCIe-7852R) controlled by a LabVIEW 2016 (National Instruments, Austin, TX) interface running the Warwick Electrochemical Scanning Probe Microscopy (WEC-SPM, www.warwick.ac.uk/electrochemistry) software.

### Data Analysis and Processing

After acquisition, the raw SECCM data were processed using the Matlab R2020a (Mathworks, Natick, MA) software package. The logarithm of *i*_surf_ data, log_10_|*i*_surf_|, was plotted *vs xy* position to create a series of time-resolved (chronoamperometry) or potential-resolved (cyclic voltammetry) images, which were combined and presented as dynamic electrochemical movies.^24,30^ The static images of electrochemical activity (*i.e.*, proton conductance), presented in the main text, were constructed by integrating |*i*_surf_| with respect to time to calculate surface charge, |*Q*_surf_|, which was plotted as log_10_|*Q*_surf_| *vs xy* position. In all electrochemical images and movies, pixels with log_10_|*i*_surf_| or log_10_|*Q*_surf_| values less than 3× the electrical noise level (calculated dynamically for each data set) are assigned a gray color and represent proton-impermeable regions of the graphene|Nafion membrane. The proton transmission site density was estimated as ≈active pixels/(total pixels × tip area), taking the area wetted by the meniscus cell during contact to be equal to the tip area of the employed pipet probe. Data plotting was carried out using the Matlab R2020 and OriginPro 2019b (OriginLab, Northampton, MA) software packages. Note that all electrochemical maps and movies are presented without any data interpolation.

## Supporting Information Available

Spatially resolved (51 × 51 pixels over a 100 × 100 μm^2^ area) voltammetric (υ = 0.1 V s^–1^, 1 cycle) movie, visualizing local proton transmission through a graphene|Nafion membrane, corresponding to Figure 2 in the main text (Movie S1) (AVI)
Spatially resolved (51 × 51 pixels over a 100 × 100 μm^2^ area) voltammetric (υ = 0.2 V s^–1^, 2 cycles) movie, visualizing local proton transmission through a graphene|Nafion membrane, corresponding to Figure 3 in the main text (Movie S2) (AVI)
Spatially resolved (49 × 29 pixels over a 120 × 70 μm^2^ area) amperometric (pulse time = 10 s) movie, visualizing local proton transmission through a “damaged area” of a graphene|Nafion membrane, corresponding to Figure 4a-i in the main text (Movie S3) (AVI)
Spatially resolved (49 × 29 pixels over a 120 × 70 μm^2^ area) amperometric (pulse time = 10 s) movie, visualizing local proton transmission through a more pristine area of a graphene|Nafion membrane, corresponding to Figure 4a-ii in the main text (Movie S4) (AVI)
Assessment of graphene as a working electrode (Figure S1); schematic of local proton conduction mechanisms (Figure S2); movie captions; colocated quasi-topographical maps, collected synchronously with electrochemical data in SECCM (Figures S3 and S4); estimation of overpotential, capacitance, and *RC* time constant (Figure S5); equivalent circuit model of ion transport through a nanopore (Figure S6); summary of reported areal conductivity values of graphene membranes (Table S1); electron microscopy images of SECCM probes (Figure S7); and XPS characterization of the Nafion and graphene|Nafion membranes (Figure S8 and Table S2) (PDF)

## Supplementary Material

nn1c05872_si_001.avi

nn1c05872_si_002.avi

nn1c05872_si_003.avi

nn1c05872_si_004.avi

## Notes

The data that support the findings of this study are available from the corresponding author, C.L.B., upon reasonable request.
