# Supplementary material for: High-Resolution Ion-Flux Imaging of Proton Transport through Graphene|Nafion Membranes
Source: ACS Nano. 2022 Mar 14;16(4):5233–45. doi: 10.1021/acsnano.1c05872 (PMC9047657; doi:10.1021/acsnano.1c05872)
Supplement: Supplementary file 5 — nn1c05872_si_005.pdf [file nn1c05872_si_005.pdf]

Supporting Information for:

High-Resolution Ion-Flux Imaging of Proton Transport through Graphene|Nafion Membranes

Cameron L. Bentley,[†,‡,] Minkyung Kang,[‡] Saheed Bukola,[§] Stephen E. Creager[§] and Patrick R. Unwin[‡,*]*

[†]School of Chemistry, Monash University, Clayton, Victoria 3800, Australia

[‡]Department of Chemistry, University of Warwick, Coventry CV4 7AL, United Kingdom

[§]Department of Chemistry, Clemson University, Clemson, South Carolina 29634, United States

*E-mail: cameron.bentley@monash.edu (C.L.B.); p.r.unwin@warwick.ac.uk (P.R.U.)

Contents

ASSESSING THE QUALITY OF GRAPHENE AS A WORKING ELECTRODE 3
LOCAL PROTON CONDUCTION MECHANISMS 5
MOVIE CAPTIONS 6
TOPOGRAPHICAL MAPS 7
OVERPOTENTIAL, CAPACITANCE AND *RC* TIME CONSTANT 9
EQUIVALENT CIRCUIT: ION TRANSPORT THROUGH A NANOPORE 11
MACROSCOPIC AREAL CONDUCTIVITY OF GRAPHENE MEMBRANES 15
ELECTRON MICROSCOPY IMAGES OF SECCM PROBES 16
XPS CHARACTERIZATION OF GRAPHENE|NAFION MEMBRANES 17
SI REFERENCES 18

ASSESSING THE QUALITY OF GRAPHENE AS A WORKING ELECTRODE

In order to assess the quality of the graphene overlayer film as a working electrode (WE), we studied the electrochemistry of the outer-sphere $FcDM^{0/+}$ (FcDM = ferrocendimethanol) process using the same dual-channel scanning electrochemical cell microscopy (SECCM) set up[1, 2] as for the proton transmission measurements. As shown in Figure S1a, in this configuration, the potential (E_{surf}) at the graphene WE surface (directly connected to the electrometer, rather than floating, as in the main text), was controlled through the applied potential (E_{app}) *via*: $E_{surf} = -(E_{app} + E_{bias}/2)$, as described in the main text. The employed dual-channel probe had a tip radius (r_{tip}) of *ca.* 100 nm (Figure S1a, inset), and during measurement, was equipped with Ag/AgCl quasi-reference counter electrodes (QRCEs) and filled with an aqueous solution of 1.3 mM FcDM + 12.5 mM HCl.

Linear-sweep voltammograms (LSVs), obtained in the SECCM format on graphene and GC WEs, shown in Figure S1c, are sigmoidal in shape, indicating (near-)steady-state behaviour,[1] with mass-transport limiting currents (i_{lim}) of ≈3.2 pA. $FcDM^{0/+}$ is an apparently ideal (Nernstian) process on both materials, evidenced by: $E_{1/2} \approx E^{0'} \approx 0.15$ V [where $E_{1/2}$ is the potential where $i = i_{lim}/2$ and; $E^{0'}$ is the formal reversible potential, estimated by macroscopic cyclic voltammetry, not shown] and; $|E_{3/4} - E_{1/4}| \approx 57$ mV (where $E_{3/4}$ and $E_{1/4}$ are the potentials where $i = 3{\cdot}i_{lim}/4$ and $i = i_{lim}/4$, respectively), satifying Tomes criterion of reversibility.[3] Assuming an upper limit of detection of the heterogeneous electron-transfer rate constant (k^0) $\approx 5{\cdot}k_m \approx D/(2r_{tip})$, where k_m is the mass-transfer coefficient [$\approx D/(10r_{tip})$ in the SECCM configuration] and D is the diffusion coefficient of FcDM ($\approx 8{\cdot}10^{-6}$ cm^2 s^{-1}); $k^0 \geq 0.04$ cm s^{-1} on both graphene and GC.[4] This effectively demonstrates that the graphene prepared as described herein is usable as a WE that supports facile electron-tunnelling, in agreement with previous reports.[5]

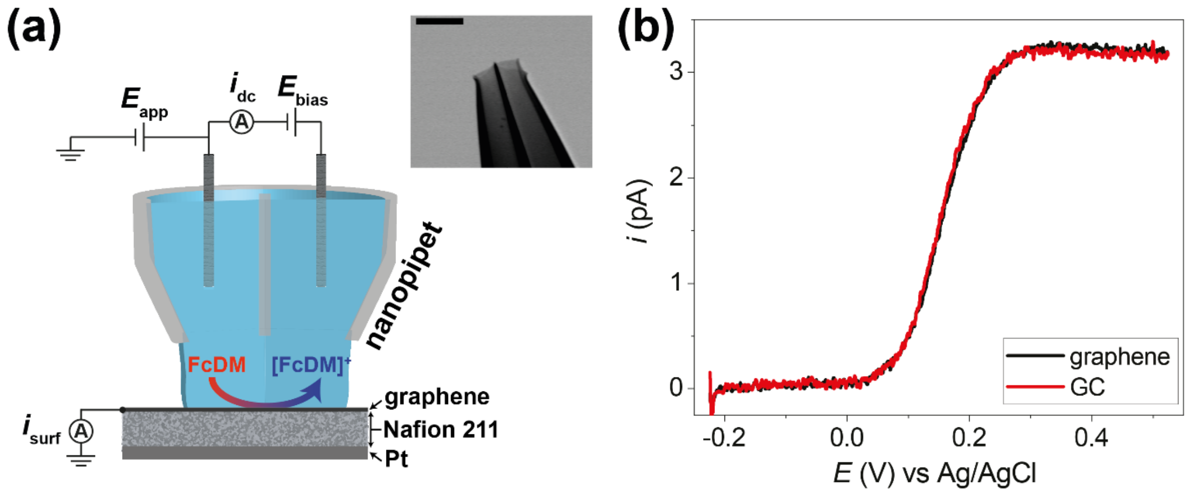

Figure S1. (a) Schematic of the SECCM set up employed to assess the quality of graphene as a working electrode. The dual-channel nanopipet probe is filled with electrolyte solution (1.3 mM FcDM + 12.5 mM HCl) and equipped with identical Ag/AgCl QRCEs. During operation, E_{bias} is applied between the QRCEs and the resulting i_{dc} is used as a feedback signal to detect meniscus-surface contact. A potential of E_{app} was applied to one of the QRCEs to control the graphene WE potential (E_{surf}), where $E_{surf} = -(E_{app} + E_{bias}/2)$ and the WE current (i_{surf}) was measured. Note that in this configuration, the Nafion membrane simply serves as a support for the graphene WE and the underlying Pt electrode was floating (*i.e.*, it was neither biased nor electrically grounded). Inset is a scanning tunnelling electron microscopy (STEM) image of the employed nanopipet tip. **(b)** Representative LSVs (voltammetric scan rate, υ = 0.2 V s^{-1}) obtained from the FcDM$^{0/+}$ process on graphene (black trace) and GC (red trace) WE substrates in the SECCM configuration [shown in (a)]. These curves are the average of 10 independent measurements carried out at randomly selected spots across the respective WE surfaces.

LOCAL PROTON CONDUCTION MECHANISMS

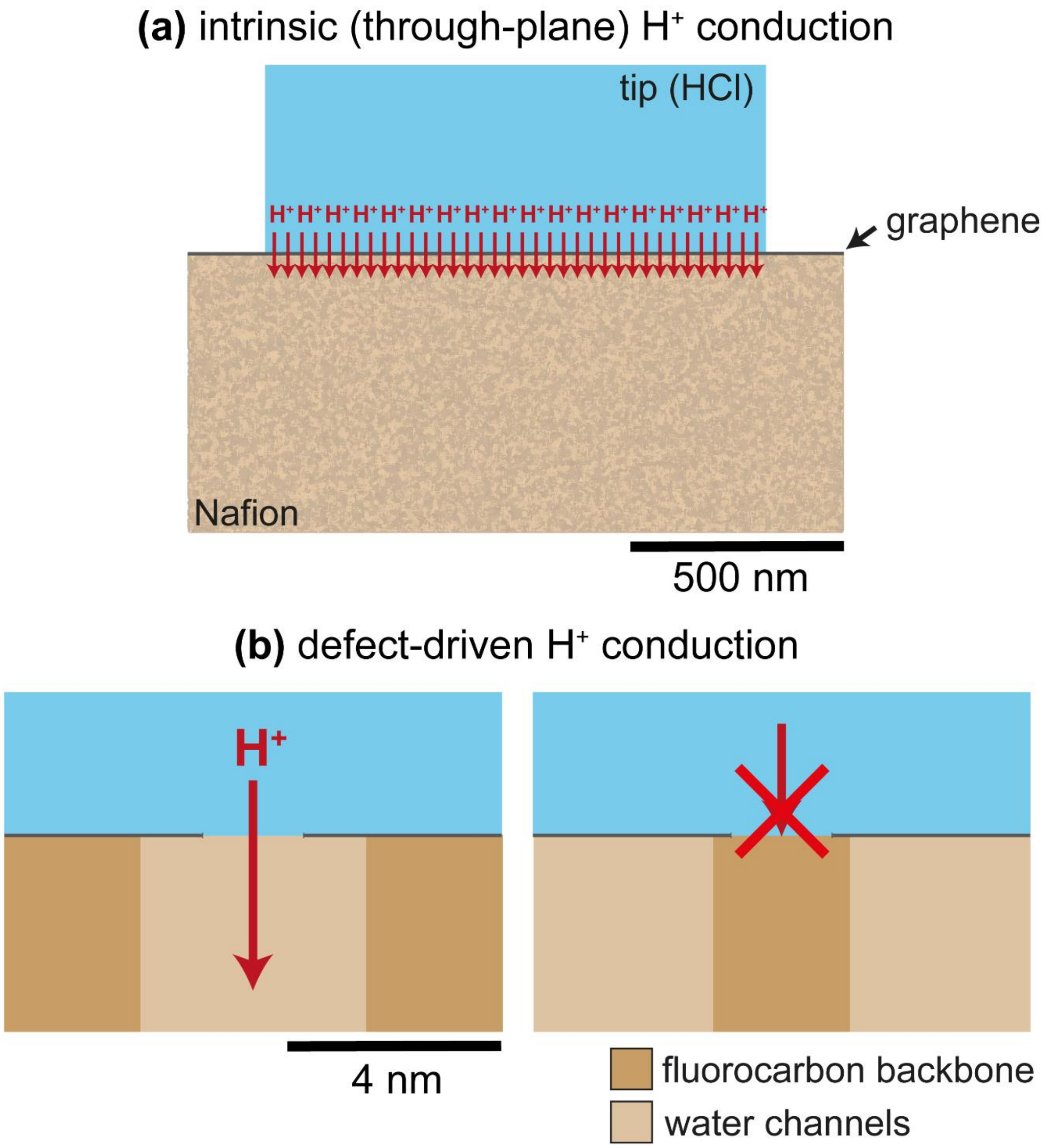

Figure S2. Scheme showing **(a)** intrinsic (through-plane) and **(b)** defect-driven proton (H^+) conduction mechanisms. Note the differing scales.

MOVIE CAPTIONS

Movie S1. Spatially-resolved electrochemical (*current−voltage, i−E*) movie (51 × 51 pixels over a 100 × 100 μm^2 area, hopping distance = 2 μm) obtained with the voltammetric (υ = 0.1 V s^{-1}, 1 cycle) hopping mode SECCM configuration (shown in the main text, Figure 1), visualizing local proton transmission through a graphene|Nafion membrane. The micropipet probe (tip area ≈ 1 μm^2) was equipped with Ag/AgCl QRCEs and filled with 0.1 M HCl. Data extracted from Movie S1 were used to construct Figure 2a in the main text. The data presented are not interpolated.

Movie S2. Spatially-resolved electrochemical (*i−E*) movie (51 × 51 pixels over a 100 × 100 μm^2 area, hopping distance = 2 μm) obtained with the voltammetric (υ = 0.2 V s^{-1}, 2 cycles) hopping mode SECCM configuration (shown in the main text, Figure 1), visualizing local proton transmission through a graphene|Nafion membrane. The micropipet probe (tip area ≈ 1 μm^2) was equipped with Ag/AgCl QRCEs and filled with 0.1 M HCl. Data extracted from Movie S2 were used to construct Figure 3a in the main text. The data presented are not interpolated.

Movie S3. Spatially-resolved electrochemical (*current−time*, *i−t*) movie (49 × 29 pixels over a 120 × 70 μm^2 area, hopping distance = 2.5 μm) obtained with the amperometric (pulse time = 10 seconds) hopping mode SECCM configuration (shown in the main text, Figure 1), visualizing local proton transmission through a "damaged area" of a graphene|Nafion membrane. The micropipet probe (tip area ≈ 2 μm^2) was equipped with Ag/AgCl QRCEs and filled with 0.1 M HCl. Data extracted from Movie S3 were used to construct Figure 4a-i in the main text. The data presented are not interpolated.

Movie S4. Spatially-resolved electrochemical (*i−t*) movie (49 × 29 pixels over a 125 × 70 μm^2 area, hopping distance = 2.5 μm) obtained with the amperometric (pulse time = 10 seconds) hopping mode SECCM configuration (shown in the main text, Figure 1), visualizing local proton transmission through a more pristine area of a graphene|Nafion membrane. The micropipet probe (tip area ≈ 2 μm^2) was equipped with Ag/AgCl QRCEs and filled with 0.1 M HCl. Data extracted from Movie S4 were used to construct Figure 4a-ii in the main text. The data presented are not interpolated.

TOPOGRAPHICAL MAPS

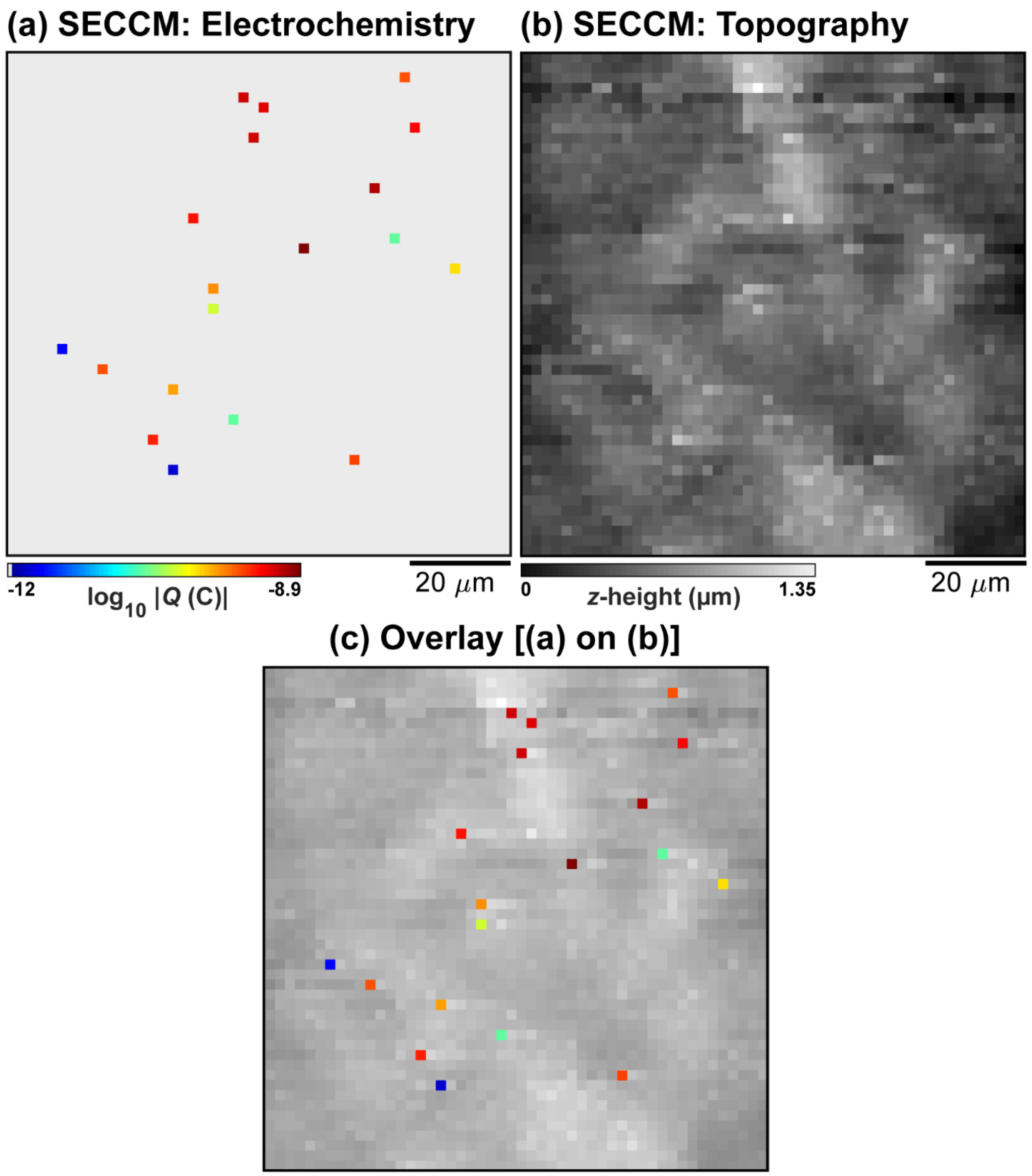

Figure S3. (a) Electrochemical activity ($\log_{10}|Q|$) and **(b)** co-located topographical maps (measured synchronously), collected over a 100 × 100 μm^2 area of a graphene|Nafion membrane, using SECCM in the voltammetric (υ = 0.1 V s^{-1}, 1 cycle, E_{surf} = −0.225 to 0.175 V *vs.* Ag/AgCl$_{QRCE}$) hopping mode configuration (hopping distance = 2 μm, 51 × 51 pixels). **(c)** Overlay of (a) on (b). Note that SI, Figure S3a is reproduced from Figure 2a in the main text.

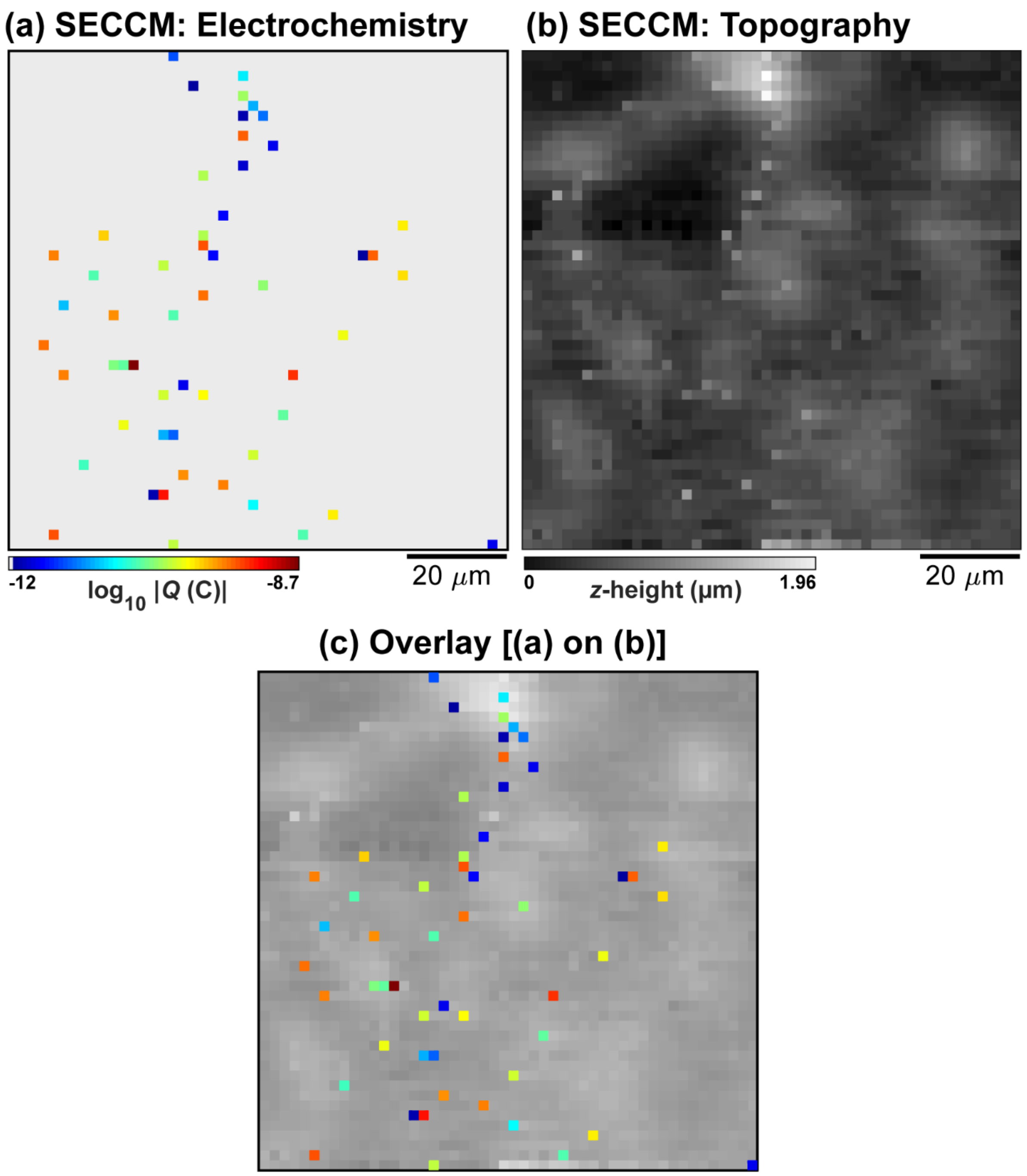

Figure S4. (a) Electrochemical activity ($\log_{10}|Q|$) and **(b)** co-located topographical maps (measured synchronously), collected over a 100 × 100 μm^2 area of a graphene|Nafion membrane, using SECCM in the voltammetric (υ = 0.2 V s^{-1}, 2 cycles, E_{surf} = −0.225 to 0.175 V *vs.* Ag/AgCl$_{QRCE}$) hopping mode configuration (hopping distance = 2 μm, 51 × 51 pixels). **(c)** Overlay of (a) on (b). Note that SI, Figure S4a is reproduced from Figure 3a in the main text.

OVERPOTENTIAL, CAPACITANCE AND *RC* TIME CONSTANT

Overpotential (η). The overpotential (η) is defined as:[3]

$$\eta = E - E_{\mathrm{eq}} \tag{S1}$$

where E_{eq} is the equilibrium potential. The hydrogen evolution reaction (HER), *i.e.*, H^+/H_2 process, by definition, possesses an E_{eq} value of 0 V *vs.* RHE (reversible hydrogen electrode), which is related to the SHE (standard hydrogen electrode) as follows:

$$E_{\mathrm{RHE}} = E_{\mathrm{SHE}} - 0.059 \cdot \mathrm{pH} \tag{S2}$$

As the HER is taking place at the Pt WE, the pH value here refers to that of the Nafion 211 membrane, which is taken to be ≈−0.1 (Refs 6-8). In 0.1 M HCl, the Ag/AgCl QRCEs are poised at:

$$E_{\mathrm{QRCE}} = E^{0\prime} - \frac{RT}{F}\ln[\mathrm{Cl^-}] \approx 0.281\ \mathrm{V}\ vs.\,\mathrm{SHE} \tag{S3}$$

Thus, −0.5 V *vs.* $Ag/AgCl_{QRCE}$ translates to ≈−0.2 V *vs.* RHE or η = 0.2 V.

Double layer capacitance (C_{dl}). C_{dl} arises from the charging of the electrical double layer at the macroscopic Pt WE and therefore is not necessarily limited to the dimensions of meniscus-surface contact. Indeed, a previous study has shown that in low-frequency AC measurements (*i.e.*, on the ms to s timescale), the nanopipet "senses" the capacitance of an electrode area that is orders-of-magnitude larger than the dimensions of the probe tip itself.[9] C_{dl} was estimated from the nonfaradaic charging current measured by landing the SECCM probe directly on the Nafion, exposed at damaged areas of the graphene|Nafion membrane; a representative cyclic voltammogram (CV) is shown in Figure S5. From the nonfaradaic current 'envelope' (marked $2 \cdot i_C$ in Figure S5), C_{dl} can be estimated as follows:

$$C_{\mathrm{dl}} = \frac{i_{\mathrm{C}}}{\upsilon} \tag{S4}$$

where υ is voltammetric scan rate. From $i_C \approx 0.2$ nA and $\upsilon = 0.1$ V s^{-1}, C is estimated to be ≈2 nF. As a rough estimate, if it is assumed that the Pt WE has a specific capacitance of 20 µF cm^{-2} (typical for a metal electrode in aqueous solution[3]), 2 nF corresponds to an electrode area of ≈10^{-4} cm^2. This means

that the area of the Pt WE that contributes to C_{dl} is *ca.* 4 orders-of-magnitude larger than that of the area of meniscus contact (*ca.* 1 – 2 μm^2), consistent with the previous report described above.[9]

***RC* time constant (*τ*).** The *RC* time constant (*τ*) is the product of the series resistance (R_{series}) and double layer capacitance (C_{dl}). As described in detail in the main text, over an active proton transmission site, $R_{series} \approx R_{pore} \approx$ 100 to 1000 MΩ, giving rise to *τ* values of 0.2 to 2 s. Assuming a simple *RC* series circuit, for a potential-step experiment, the non-faradaic current (i_{nf}) decays exponentially with *t* as follows:

$$i_{\mathrm{nf}} = \frac{E}{R} e^{-t/\tau} \qquad (S5)$$

Thus, i_{nf} drops to 5% of its initial value at $t = 3\tau$, meaning it can be effectively neglected at $t > 6$ s (assuming $\tau = 2$ s).

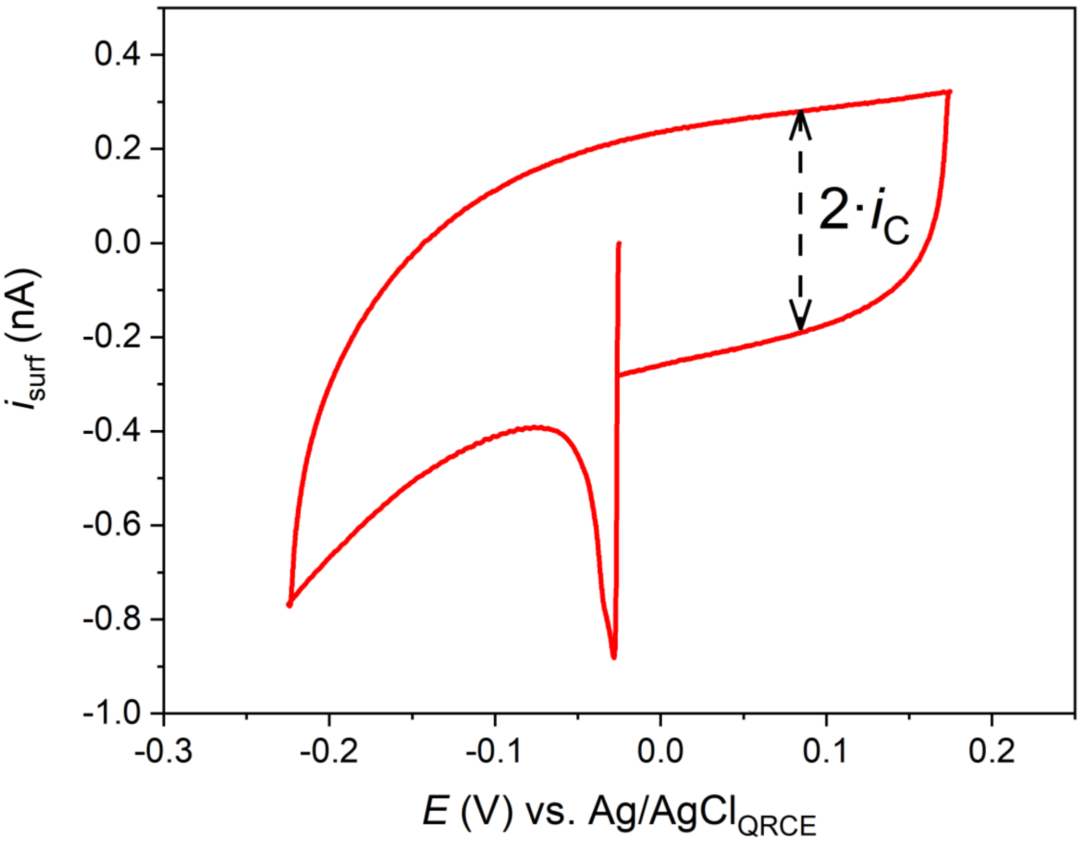

Figure S5. Representative CV (υ = 0.1 V s^{-1}, 1 cycle, E_{surf} = −0.225 to 0.175 V *vs.* Ag/AgCl$_{QRCE}$), obtained in the SECCM format by landing the micropipet probe directly on the Nafion 211 film (exposed at a damaged area of the graphene|Nafion membrane). This curve is the average of 10 independent measurements. These data were collected with a dual-channel micropipet probe of tip area ≈1 μm^2, equipped with Ag/AgCl QRCEs and filled with 0.1 M HCl.

EQUIVALENT CIRCUIT: ION TRANSPORT THROUGH A NANOPORE

Analogizing proton conduction through local transmission "sites" on the graphene|Nafion membranes to ion transport through an atomically-thin, cylindrical nanopore,[10, 11] the equivalent circuit model[12, 13] schematized in Figure S6 was derived.

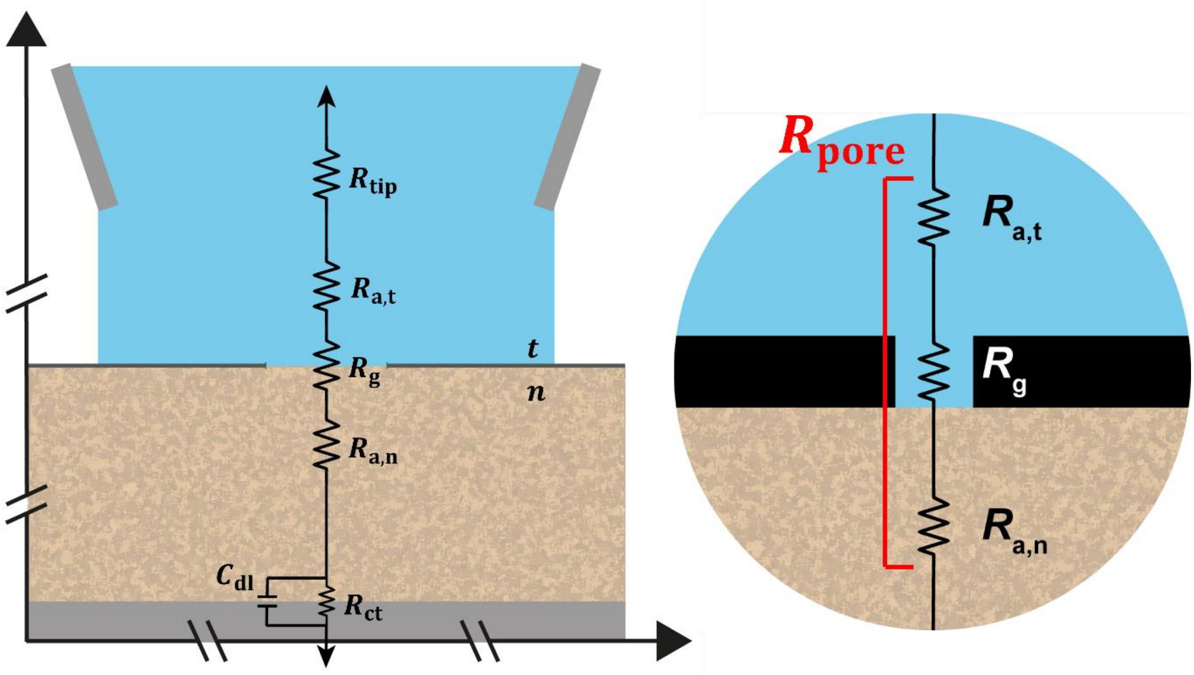

Figure S6. Equivalent circuit representing local proton transmission through graphene|Nafion membranes. The transmission site is analogized to a cylindrical nanopore (zoomed in on right) in the graphene overlayer film. R_{tip} = tip resistance; R_a = access resistance; R_g = geometric resistance; R_{pore} = pore resistance; R_{ct} = charge-transfer resistance and; C_{dl} = double layer capacitance. Note that R_{pore} = $R_{a,t}$ + R_g + $R_{a,n}$ (shown on right). The subscripts *t* and *n* refer to the tip and Nafion sides of the graphene film, respectively.

Beginning from the top of Figure S6, R_{tip} is the resistance of the micropipet tip, which is known to depend on the inner cone angle (β) and tip radius (r_t) according to:

$$R_{\mathrm{tip}} \approx \frac{\rho}{\pi r_{\mathrm{t}} \tan\beta} + \frac{\rho}{4 r_{\mathrm{t}}} \quad \text{(S6)}$$

where ρ is the solution resistivity. In practice, R_{tip} can be estimated from using Ohm's law, by measuring an current−voltage curve in the scanning ion conductance microscopy (SICM)

configuration.[14] Alternatively, when using a dual-channel probe, R_{tip} can be readily estimated as follows:

$$R_{\text{tip}} \approx \frac{E_{\text{bias}}}{i_{\text{dc}}} \tag{S7}$$

where E_{bias} is the bias voltage that is applied between the Ag/AgCl quasi-reference counter electrodes (QRCEs) located in the two channels and i_{dc} is the ion conductance current that flows through the meniscus located at the end of the probe.

R_a is the access resistance, which refers to the resistance through solution from electrodes (*i.e.*, QRCE and WE) to pore aperture, and depends on the pore radius (assuming a circular pore of radius, r_p) as follows:[10, 11]

$$R_{\text{a,t}} = \frac{\rho_{\text{t}}}{4r_{\text{p}}} \tag{S8}$$

$$R_{\text{a,n}} = \frac{\rho_{\text{n}}}{4r_{\text{p}}} \tag{S9}$$

Note that the subscripts *t* and *n* refer to the *tip* (*i.e.*, 0.1 M HCl) and *Nafion* sides of the graphene film, respectively. R_g is the geometric resistance, which is proportional to the length (L_p) and inversely proportional to the area (πr_p^2) of nanopore, as follows:[10, 11]

$$R_{\text{g}} = \frac{\rho_{\text{p}} L_{\text{p}}}{\pi r_{\text{p}}^2} \tag{S10}$$

Note that the subscript *p* signifies properties of the pore itself. Assuming $\rho_t = \rho_n = \rho_p = \rho$ (*vide infra*), consolidating Eqs. (S8) – (S10) gives the following (Figure S6):

$$R_{\text{pore}} = \frac{\rho L_{\text{p}}}{\pi \text{r}_{\text{p}}^2} + \frac{\rho}{2r_{\text{p}}} \tag{S11}$$

where R_{pore} is the pore resistance. From Eq. (S11), pores with a high aspect ratio (*i.e.*, $r_p < L_p$) give a pore-dominated response (*i.e.*, $R_g > R_p$), whereas pores with a low aspect ratio ($r_p > L_p$) give an access-dominated response (*i.e.*, $R_g < R_p$). Monolayer graphene is atomically thin, with L_p estimated to be in the ≈0.34 nm (*i.e.*, van der Waals diameter of carbon atoms) to ≈1 nm range,[10] taken to be ≈0.6 nm,

herein.[15] Thus, for atomic-scale proton transmission sites ($r_p \approx L_p$), both R_g and R_p contribute to overall pore resistance (R_{pore}), whereas for lower aspect ratio or nanoscale sites ($r_p > L_p$), $R_{pore} \approx R_a$.

R_{ct} is the charge transfer resistance (or activation resistance), which scales inversely with the heterogeneous electron-transfer rate constant associated with the Faradaic charge-transfer reaction taking place at the Pt WE [*e.g.*, oxygen reduction reaction (ORR), HER *etc.*]. R_{ct} is in parallel with C_{dl}, which is the double layer capacitance, arising from the charging of the electrical double layer on the macroscopic Pt WE.[3] Note that because the Ag/AgCl process is kinetically facile, and the potential of the QRCE is fixed during measurement,[16] R_{ct} and C_{dl} on the Ag/AgCl QRCEs (not shown in Scheme 1) can be neglected.[3]

In principle, the pore geometry (*i.e.*, r_p) can be estimated if R_{pore} is known, *i.e.*, rearranging Eq. (S11) gives:

$$r_{\mathrm{p}} = \frac{\pi\rho + \sqrt{\pi^2\rho^2 + 16\pi \cdot R_{\mathrm{pore}} \cdot \rho \cdot L_{\mathrm{p}}}}{4\pi \cdot R_{\mathrm{pore}}} \qquad \textbf{(S12)}$$

In practice, R_{pore} can only be estimated simply from the series resistance (R_{series}) under conditions where the contributions from C_{dl} and R_{ct} are negligible (i.e., $R_{series} \approx R_{tip} + R_{pore}$; see Figure S6). By applying a potential-step (*e.g.*, main text, Figure 4) waveform, rather than a potential-sweep (*e.g.*, main text, Figures 2 and 3), the capacitive contribution to the measured surface current (i_{surf}) can be effectively neglected after a time of 3τ (*i.e.*, where the double layer charging current drops to 5% of its initial value[3]), which is >6 seconds, assuming τ = 2 s (*vide supra*). R_{ct} can be effectively neglected under conditions of facile electron-transfer, which can be readily achieved herein by only considering the kinetically facile HER (rather than the kinetically sluggish ORR) at a strongly driving overpotential (η). Indeed, it is the rapid electron-transfer kinetics of the H^+/H_2 process at Pt electrodes that enables them to be used in conventional electrochemical hydrogen pump cells to quantify (bulk) proton transport in proton exchange membrane fuel cells.[17]

Thus, during a potential-step experiment at $t > 6$ s and a strong driving (over)potential of η = 0.2 V (*ca.* −0.5 V *vs.* Ag/AgCl, calculation detailed above), R_{series} reduces to:

$$R_{\text{series}} \approx R_{\text{tip}} + R_{\text{pore}} \approx \frac{\eta}{i_{\text{surf}}} \tag{S13}$$

and rearranging for R_{pore}:

$$R_{\text{pore}} \approx \frac{\eta}{i_{\text{surf}}} - R_{\text{tip}} \tag{S14}$$

Thus, as discussed in the main text, R_{pore} can be readily estimated from i_{surf} and R_{tip} [Eq. (S7)], allowing the pore geometry (r_p) to be estimated.

As noted above, in the derivation of Eq. (S11), was assumed that $\rho_t = \rho_n = \rho_p = \rho$. While the bulk conductivities of 0.1 M HCl (≈0.04 S cm^{-1}, Ref 18) and Nafion (≈0.02 – 0.06 S cm^{-1}, Ref 19) are comparable (*i.e.*, $\rho_t = \rho_n$), the local conductivity of the latter is structure-dependent on the nanoscale (*e.g.*, see Figure S2) and the environment inside the pore (*i.e.*, ρ_p) is also likely to be significantly different to the surrounding solutions. Given this, along a number of other assumptions [*e.g.*, assuming continuum ion transport and ignoring the effects of ion dehydration, interactions with surface charge(s) within the pore and structural fluctuations (flexibility) in the membrane[10]], the pore geometries (r_p) estimated in the main text from Eq. (S12) should be taken *cum grano salis*.

MACROSCOPIC AREAL CONDUCTIVITY OF GRAPHENE MEMBRANES

Table S1. Reported areal conductivity values of graphene membranes

G/A (S cm^{-2})	Preparation method / ≈membrane area	Reference	Equivalent defect density (defects µm^{-2})†
0.003	Exfoliated graphene / ≈µm^2	20	0.005
0.004	CVD graphene / ≈µm^2	21	0.007
≈0.09	CVD graphene-on-Nafion / ≈cm^2	22	≈0.2
≈0.6	H_2 plasma-treated graphene / ≈µm^2	21	≈1
≈1	Disordered graphene / ≈µm^2	23	≈2
≈1	CVD graphene-on-Nafion / ≈cm^2	24	≈2
≈30	CVD graphene-on-Nafion / ≈cm^2	25	≈50

†defects µm^{-2} = areal conductivity (S cm^{-2}) · 170·10^6 (Ω · defect) / 10^8 (cm^2 µm^{-2})

ELECTRON MICROSCOPY IMAGES OF SECCM PROBES

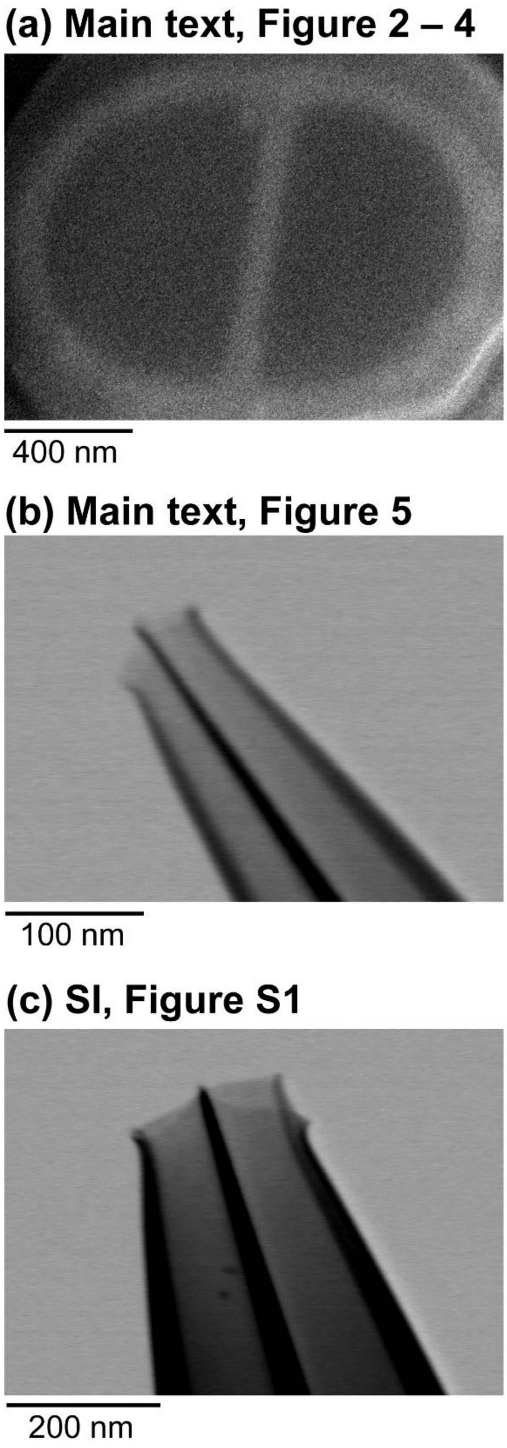

Figure S7. Representative electron microscopy images of the pipet probes used in **(a)** main text, Figures 2 – 4; **(b)** main text, Figure 5 and; **(c)** SI, Figure S1. (a) was taken in scanning electron microscopy (SEM) mode, and (b)/(c) were taken in scanning transmission electron microscopy (STEM) mode on a GeminiSEM 500 system.

XPS CHARACTERIZATION OF GRAPHENE|NAFION MEMBRANES

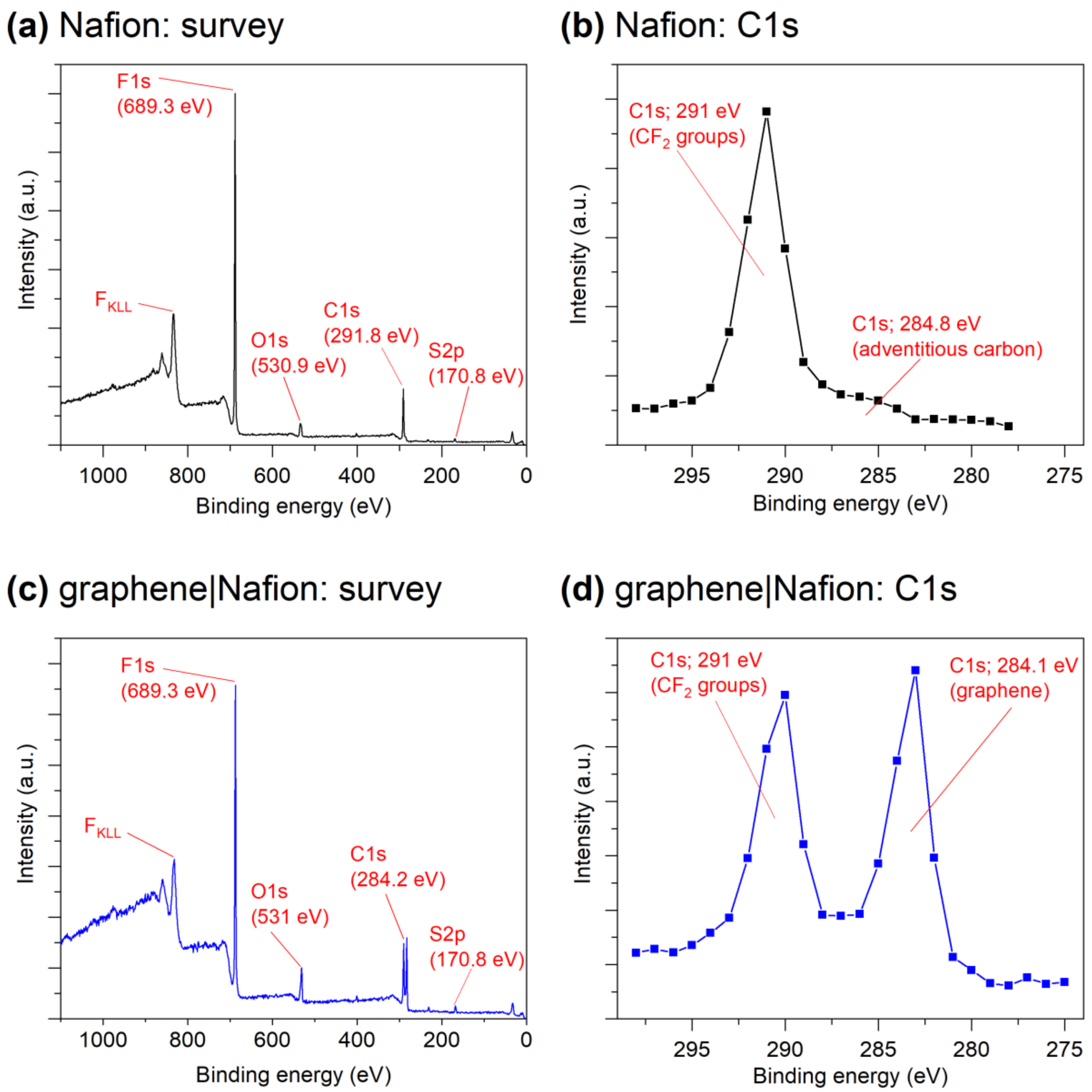

Figure S8. X-ray photoelectron spectroscopy (XPS) spectra obtained from Nafion [**(a)** survey and **(b)** C1s] and graphene|Nafion [**(c)** survey and **(d)** C1s] membranes.

Table S2. Atomic composition of Nafion and graphene|Nafion membranes, quantified by XPS.

**element**	**atomic %**	
	Nafion	graphene|Nafion
F1s	57.7	33
C1s	33.8	58
O1s	7.5	8
S2p	1	0.9

SI REFERENCES

1. Snowden, M. E.; Güell, A. G.; Lai, S. C. S.; McKelvey, K.; Ebejer, N.; O'Connell, M. A.; Colburn, A. W.; Unwin, P. R., Scanning Electrochemical Cell Microscopy: Theory and Experiment for Quantitative High Resolution Spatially-Resolved Voltammetry and Simultaneous Ion-Conductance Measurements. *Anal. Chem.* **2012,** *84* (5), 2483-2491.
2. Ebejer, N.; Güell, A. G.; Lai, S. C. S.; McKelvey, K.; Snowden, M. E.; Unwin, P. R., Scanning Electrochemical Cell Microscopy: A Versatile Technique for Nanoscale Electrochemistry and Functional Imaging. *Annu. Rev. Anal. Chem.* **2013,** *6*, 329-351.
3. Bard, A. J.; Faulkner, L. R., *Electrochemical Methods : Fundamentals and Applications*. 2nd ed.; Wiley: New York, 2001; p 833.
4. Daviddi, E.; Chen, Z.; Massani, B. B.; Lee, J.; Bentley, C. L.; Unwin, P. R.; Ratcliff, E. L., Nanoscale Visualization and Multiscale Electrochemical Analysis of Conductive Polymer Electrodes. *ACS Nano* **2019,** *13* (11), 13271-13284.
5. Güell, A. G.; Cuharuc, A. S.; Kim, Y.-R.; Zhang, G.; Tan, S.-y.; Ebejer, N.; Unwin, P. R., Redox-Dependent Spatially Resolved Electrochemistry at Graphene and Graphite Step Edges. *ACS Nano* **2015,** *9* (4), 3558-3571.
6. Umeda, M.; Sayama, K.; Maruta, T.; Inoue, M., Proton Activity of Nafion 117 Membrane Measured from Potential Difference of Hydrogen Electrodes. *Ionics* **2013,** *19* (4), 623-627.
7. Spry, D. B.; Fayer, M. D., Proton Transfer and Proton Concentrations in Protonated Nafion Fuel Cell Membranes. *J. Phys. Chem. B* **2009,** *113* (30), 10210-10221.
8. Seger, B.; Vinodgopal, K.; Kamat, P. V., Proton Activity in Nafion Films: Probing Exchangeable Protons with Methylene Blue. *Langmuir* **2007,** *23* (10), 5471-5476.
9. Shkirskiy, V.; Kang, M.; McPherson, I. J.; Bentley, C. L.; Wahab, O. J.; Daviddi, E.; Colburn, A. W.; Unwin, P. R., Electrochemical Impedance Measurements in Scanning Ion Conductance Microscopy. *Anal. Chem.* **2020,** *92* (18), 12509-12517.
10. Sahu, S.; Zwolak, M., Colloquium: Ionic Phenomena in Nanoscale Pores through 2D Materials. *Rev. Mod. Phys.* **2019,** *91* (2), 021004.
11. Hyun, C.; Rollings, R.; Li, J., Probing Access Resistance of Solid-State Nanopores with a Scanning-Probe Microscope Tip. *Small* **2012,** *8* (3), 385-392.
12. Zhang, Z.-Y.; Deng, Y.-S.; Tian, H.-B.; Yan, H.; Cui, H.-L.; Wang, D.-Q., Noise Analysis of Monolayer Graphene Nanopores. *Int. J. Mol.* **2018,** *19* (9), 2639.
13. Kowalczyk, S. W.; Grosberg, A. Y.; Rabin, Y.; Dekker, C., Modeling the Conductance and DNA Blockade of Solid-State Nanopores. *Nanotechnology* **2011,** *22* (31), 315101.

14. Perry, D.; Momotenko, D.; Lazenby, R. A.; Kang, M.; Unwin, P. R., Characterization of Nanopipettes. *Anal. Chem.* **2016,** *88* (10), 5523-5530.

15. Garaj, S.; Hubbard, W.; Reina, A.; Kong, J.; Branton, D.; Golovchenko, J. A., Graphene as a Subnanometre Trans-Electrode Membrane. *Nature* **2010,** *467* (7312), 190-3.

16. Bentley, C. L.; Perry, D.; Unwin, P. R., Stability and Placement of Ag/AgCl Quasi-Reference Counter Electrodes in Confined Electrochemical Cells. *Anal. Chem.* **2018,** *90* (12), 7700-7707.

17. Huth, A.; Schaar, B.; Oekermann, T., A "Proton Pump" Concept for the Investigation of Proton Transport and Anode Kinetics in Proton Exchange Membrane Fuel Cells. *Electrochim. Acta* **2009,** *54* (10), 2774-2780.

18. Creager, S., 3 - Solvents and Supporting Electrolytes. In *Handbook of Electrochemistry*, Zoski, C. G., Ed. Elsevier: Amsterdam, 2007; pp 57-72.

19. Peron, J.; Mani, A.; Zhao, X.; Edwards, D.; Adachi, M.; Soboleva, T.; Shi, Z.; Xie, Z.; Navessin, T.; Holdcroft, S., Properties of Nafion® NR-211 Membranes for PEMFCs. *J. Membr. Sci.* **2010,** *356* (1-2), 44-51.

20. Hu, S.; Lozada-Hidalgo, M.; Wang, F. C.; Mishchenko, A.; Schedin, F.; Nair, R. R.; Hill, E. W.; Boukhvalov, D. W.; Katsnelson, M. I.; Dryfe, R. A. W.; Grigorieva, I. V.; Wu, H. A.; Geim, A. K., Proton Transport through One-Atom-Thick Crystals. *Nature* **2014,** *516*, 227.

21. Chaturvedi, P.; Vlassiouk, I. V.; Cullen, D. A.; Rondinone, A. J.; Lavrik, N. V.; Smirnov, S. N., Ionic Conductance through Graphene: Assessing Its Applicability as a Proton Selective Membrane. *ACS Nano* **2019,** *13* (10), 12109-12119.

22. Lozada-Hidalgo, M.; Zhang, S.; Hu, S.; Esfandiar, A.; Grigorieva, I. V.; Geim, A. K., Scalable and Efficient Separation of Hydrogen Isotopes Using Graphene-Based Electrochemical Pumping. *Nat. Comm.* **2017,** *8* (1), 15215.

23. Griffin, E.; Mogg, L.; Hao, G.-P.; Kalon, G.; Bacaksiz, C.; Lopez-Polin, G.; Zhou, T. Y.; Guarochico, V.; Cai, J.; Neumann, C.; Winter, A.; Mohn, M.; Lee, J. H.; Lin, J.; Kaiser, U.; Grigorieva, I. V.; Suenaga, K.; Özyilmaz, B.; Cheng, H.-M.; Ren, W., *et al.*, Proton and Li-Ion Permeation through Graphene with Eight-Atom-Ring Defects. *ACS Nano* **2020,** *14* (6), 7280-7286.

24. Bukola, S.; Beard, K.; Korzeniewski, C.; Harris, J. M.; Creager, S. E., Single-Layer Graphene Sandwiched between Proton-Exchange Membranes for Selective Proton Transmission. *ACS Appl. Nano Mater.* **2019,** *2* (2), 964-974.

25. Bukola, S.; Liang, Y.; Korzeniewski, C.; Harris, J.; Creager, S., Selective Proton/Deuteron Transport through Nafion|Graphene|Nafion Sandwich Structures at High Current Density. *J. Am. Chem. Soc.* **2018,** *140* (5), 1743-1752.
